# Scoping Review of Sexual and Gender Minority Health Research in Ireland

**DOI:** 10.1111/jan.70201

**Published:** 2025-09-16

**Authors:** John P. Gilmore, Tonda L. Hughes, Sean Kearns, Laurie A. Drabble, Diarmuid Stokes, Siobhán D. Thomas, Chris Noone, Lauren Bochicchio

**Affiliations:** ^1^ School of Nursing, Midwifery and Health Systems University College Dublin Dublin Ireland; ^2^ Center for Sexual and Gender Minority Health Research, School of Nursing Columbia University New York New York USA; ^3^ Center for Applied Research in Human Services, College of Health and Human Sciences San José State University San José California USA; ^4^ Library Services University College Dublin Dublin Ireland; ^5^ School of Applied Psychology University College Cork Cork Ireland; ^6^ School of Psychology University of Galway Galway Ireland

**Keywords:** health inequalities, Ireland, LGBTQI+, mental health, minority stress, nursing, sexual and gender minority health, sexual health, transgender healthcare

## Abstract

**Aim:**

To map existing sexual and gender minority (SGM) health research in Ireland, identify gaps in literature and outline priorities for future research and healthcare. SGM is an umbrella term that includes people who identify as lesbian, gay, bisexual, transgender, queer or intersex and is sometimes abbreviated as LGBTQI+.

**Design:**

A scoping review of peer‐reviewed studies published between 2014 and 2024.

**Methods:**

The review followed Joanna Briggs Institute (JBI) guidelines and PRISMA‐ScR framework for scoping reviews. Articles were identified through systematic database searches and screened independently by reviewers.

**Data Sources:**

PubMed, PsycINFO, CINAHL and Embase were searched for articles published between January 2014 and April 2024. Sixty studies met inclusion criteria.

**Results:**

The review highlighted a disproportionate focus on gay, bisexual and other men who have sex with men (gbMSM), particularly regarding HIV and sexual health. Mental health research revealed high levels of anxiety, depression and suicidality, largely attributed to minority stress and systemic discrimination. Transgender health studies documented barriers to accessing gender‐affirming care and mental health services. Few studies explored experiences of sexual minority women, older SGM individuals or intersex people. Intersectional perspectives on race, disability and socio‐economic status were notably absent.

**Conclusion:**

SGM health research in Ireland reflects significant progress in documenting disparities in mental and sexual health. However, there is a lack of representation for some groups. There is also limited attention to intersectionality. Systematic gaps in sexual orientation and gender identity (SOGI) data impede targeted policymaking and service delivery.

**Implications for the Profession and/or Patient Care:**

Findings underscore the need for inclusive, culturally competent healthcare services, better integration of SGM health topics into nursing education, and community‐centred interventions. Addressing structural barriers and improving provider competence can enhance equitable healthcare access for SGM populations.

**Impact:**

This review addresses the fragmented state of SGM health research in Ireland, highlighting gaps in representation and systemic issues.

**No Patient or Public Contribution:**

Authorship includes individuals from various sexual and gender minority communities.

## 1 Introduction

Research related to the health of people who identify as lesbian, gay, bisexual, transgender, queer or intersex (LGBTQI) has gained increasing importance globally due to growing evidence of the significant health inequalities faced by the population groups (Abboud et al. 2022; Bränström et al. 2024, Cheng et al. 2022; Ferrer et al. 2022; Harkins and Hoffmann 2024; Hughes et al. 2020a, 2023; Matthews et al. 2018; Medina‐Martínez et al. 2021; Mulé 2015; Reisner et al. 2016; Zeeman et al. 2019). These groups are now commonly referred to as sexual and gender minorities (SGM), especially in academic and scholarly contexts. Despite progress in legal protections and social acceptance in many countries, SGM health disparities persist, driven by factors such as discrimination, social exclusion and systemic bias in healthcare settings (Fish et al. 2021).

Regardless of country or region, studies consistently show that, compared to their heterosexual counterparts, SGM individuals experience higher rates of mental health concerns, substance use disorders and certain physical health conditions (Bränström et al. 2024; Harkins and Hoffmann 2024; Hughes et al. 2016, 2020; Zeeman et al. 2019). Mental health conditions such as depression, anxiety and suicidal ideation and attempts are more prevalent in SGM communities, often exacerbated by experiences of minority stress such as discrimination and internalised homonegativity (Bränström et al. 2024; Hughes et al. 2020a; Medina‐Martínez et al. 2021). Harmful substance use, including tobacco, alcohol and drug abuse, is also more common among SGM individuals, especially among sexual minority women (SMW) (Hughes et al. 2016, 2020b; Moagi et al. 2021). Additionally, some SGM subgroups, such as gay and bisexual men, face heightened risks of HIV transmission and other sexually transmitted infections (STIs) (Zeeman et al. 2019), while transgender and gender diverse individuals frequently report challenges in accessing appropriate healthcare, including gender‐affirming treatments and preventive care (Reisner et al. 2016).

In Ireland, these international trends are mirrored by research highlighting the ongoing challenges faced by SGM individuals in accessing equitable healthcare (Higgins, Doyle, et al. 2016). An important feature of much of this research is collaboration between academics and community organisations. Indeed, SGM health research in Ireland has historically been driven by the community. These organisations have published reports describing health‐related research on the themes of mental health (Higgins, Doyle, et al. 2016; Higgins et al. 2024; Mayock et al. 2009; Mental Health Reform 2022); ageing (Higgins et al. 2011); migration (Noone et al. 2018), access to transgender healthcare (Mallon 2024; Quilty and Kennedy 2024), sexual health (Brady et al. 2020; Carroll et al. 2002; Casey et al. 2019; Gay Health Action 1989; Gilmore et al. 2023; McCartney 2009; McCartney et al. 2009; McNeil et al. 2013; O'Donnell et al. 2016; Quinlan et al. 1992), drug use (Sarma 2007) and COVID‐19 (Belong To Youth Services 2021; LGBT Ireland, NXF and GCN 2020; Witzel et al. 2022).

While community reports play a vital role in highlighting the health issues faced by SGM people, their influence on healthcare policy and practice in Ireland appears limited. This is largely due to a policy‐making culture that prioritises peer‐reviewed academic evidence, leaving the contributions of community‐generated knowledge under‐recognised and under‐utilised.

Although recent legislative reforms, such as the Gender Recognition Act (2015) and Marriage Equality Act (Thirty‐fourth Amendment of the Constitution (Marriage Equality) 2015) have improved the legal standing of SGM people, significant barriers to health equity remain, especially in healthcare settings. Discrimination, lack of cultural competence among healthcare providers, and assumptions stemming from cis heteronormativity continue to hinder SGM individuals from receiving the care they need (Gilmore 2023). To our knowledge, this review is the first comprehensive synthesis of academic health‐related research focused on SGM health in Ireland. Among its goals is to provide a foundation for more targeted research on these understudied and underserved population groups in Irish health research and healthcare.

### 1.1 Review Objectives

In light of these goals, we conducted a scoping review of the peer‐reviewed literature focusing on the health of SGM people in Ireland. A scoping review is a type of evidence synthesis that maps the breadth of literature on a topic, identifying key concepts, sources and gaps (Tricco et al. 2018).

Specific objectives of this scoping review were to:

1. Map the existing body of published research in the field of SGM health research in Ireland.
2. Identify gaps in existing literature on SGM health research in terms of both thematic and demographic makeup.
3. Identify priorities for future health research and healthcare provision for SGM people in Ireland

## 2 Materials and Methods

The protocol for this scoping review was registered prospectively in Open Science Framework (OSF) (https://osf.io/uvqj9/). We used the Joanna Briggs Institute (JBI) guidance for scoping reviews to guide the review (Peters et al. 2020). In line with JBI guidance, a structured data extraction tool was developed to capture key information from selected studies, including authors, year of publication, study location, aims, population characteristics, methods and key findings. This approach ensured consistency and facilitated the mapping of evidence.

The PRISMA‐ScR guidelines further informed the process by providing a framework for transparent reporting, including the clear documentation of how studies were selected, how data were charted, and how the synthesis was conducted. Together, these frameworks supported a systematic and replicable extraction process, enabling a comprehensive overview of the available evidence. For reporting the results, we adhered to the Preferred Reporting Items for Systematic Reviews and Meta‐Analyses extension for Scoping Reviews (PRISMA‐ScR) (Tricco et al. 2018).

### 2.1 Inclusion Criteria

The review included empirical peer‐reviewed journal articles published in English from 2014 to 2024 reporting findings related to physical or mental health among SGM individuals in Ireland. This 10‐year period reflected significant social and policy change in Ireland, including the introduction of marriage equality and gender recognition legislation, both in 2015. Studies with heterosexual or cisgender individuals in the broader population were included if data were analysed in such a way that findings specific to SGM participants were reported. Studies conducted outside of Ireland, such as those with multi‐country samples, were included if findings for the Irish sample were specifically reported. Literature review articles and commentaries were excluded.

A broad understanding of health was adopted in line with the World Health Organization's definition, which recognises health as not merely the absence of disease or infirmity, but a state of complete physical, mental and social wellbeing (World Health Organization, n.d.; para. 14). This approach allows for a more holistic consideration of the social determinants of health and the unique experiences of SGM individuals, beyond narrow biomedical frameworks.

### 2.2 Search and Selection Strategy

We searched four databases to identify relevant studies published between January 2014 and April 2024. These included PubMed (pubmed.gov), PsycInfo (EBSCO), CINAHL (EBSCO) and Embase (embase.com). All authors have experience in conducting scoping reviews; search terms were developed in consultation with a specialist librarian and were entered into the advanced search field in each database (Supplementary File S1). The search yielded 1455 records. Duplicate records (*n* = 222) were removed in the citation manager, EndNote (version X9). The remaining 1235 records were then imported into Covidence for title/abstract screening and full‐text screening. An example of search terms can be seen in Table 1.

**TABLE 1:** Example of search terms.

Population		Concept		Context
“Sexual and Gender Minorities”[Mesh] OR “Homosexuality”[Mesh] OR “Bisexuality”[Mesh] OR “Transsexualism”[Mesh] OR “Gender Identity”[Mesh] OR “Gender Dysphoria”[Mesh] OR “Health Services for Transgender Persons”[Mesh] OR “Disorders of Sex Development”[Mesh] OR “gender nonconform*” OR“gender non‐conform*” OR “trans female*” OR “trans male*” OR “trans man” OR “trans men” OR “trans women” OR “trans woman” OR “trans population*” OR “trans patient*” OR “trans participant*” OR “trans subject*” OR “trans individual*” OR “trans people” OR “trans person*” OR “trans youth*” OR agender OR bicurious OR bigender* OR bisexual* OR “cross sex” OR crossgender OR “DSD” OR gay OR gays OR “gender change” OR “gender crossing” OR “gender dysphori*” OR “gender fluid*” OR “gender identit*” OR “gender incongruen*” OR “gender minorit*” OR “gender neutral” OR “gender queer” OR “gender transition*” OR “gender varian*” OR genderless OR genderqueer* OR “GLB” OR “GLBQ” OR “GLBs” OR “GLBT” OR “GLBTQ” OR heteroflexible OR homosexual* OR intersex* OR lesbian* OR lesbigay* OR “LGB” OR “LGBQ” OR “LGBS” OR “LGBT*” OR “men who have sex with men” OR “mostly‐heterosexual” OR “MSM” OR “MSMW” OR nonbinary OR “non‐binary” OR nonheterosexual* OR non‐heterosexual* OR queer OR queers OR “same gender loving” OR “same sex couple*” OR “same sex relations*” OR “same‐sex attract*” OR “sexual identit*” OR “sexual minorit*” OR “sexual orientation*” OR “sexual preference*” OR “SGM” OR “third gender*” OR transex* OR transfeminine OR transgender* OR transman OR transmen OR transmasculine OR transsex* OR “trans‐sex*” OR “trans‐spectrum” OR transwoman OR transwomen OR “twospirit*” OR “women loving women” OR “women who have sex with women” OR “WSW” OR “WSWM” OR “LGBTQ” OR “mostly heterosexual women” OR “same sex” OR “same gender” OR “sexual and gender minorities” OR “sexual and gender minority” OR “TGNB” OR “TGNC” OR neutrois OR “gender euphori*” OR rainbow* OR “rainbow communities” OR “gender continuum” OR “gender spectrum” OR pansexual OR transvestite OR dyke	AND	“Stress, Psychological”[Mesh] OR “Behavior, Addictive”[Mesh] OR “Adjustment Disorders”[Mesh] OR “Mental Disorders”[Mesh] OR “Agoraphobia”[Mesh] OR “Substance‐Related Disorders”[Mesh] OR “Personality Disorders”[Mesh] OR “Burnout, Psychological”[Mesh] OR “Combat Disorders”[Mesh] OR “Compassion Fatigue”[Mesh] OR “Adaptation, Psychological”[Mesh] OR “Counselling”[Mesh] OR “Mental Health Services”[Mesh] OR “Psychiatry”[Mesh] OR “Psychotherapy”[Mesh] OR “Cyclothymic Disorder”[Mesh] OR “Depression”[Mesh] OR “Depressive Disorder”[Mesh] OR “Psychological Distress”[Mesh] OR “Hydrocodone”[Mesh] OR “Ketamine”[Mesh] OR “Methamphetamine”[Mesh] OR “Morphine”[Mesh] OR “Opiate Alkaloids”[Mesh] OR“acute stress” OR Addict OR addiction OR addictive OR addicts OR “adjustment disorder*” OR “affective disorder*” OR agoraphobi* OR alcoholism anorexi* OR “anti‐social personality disorder*” OR “antisocial personality disorder*” OR “anxiety disorder*” OR “avoidant personality disorder*” OR “binge drink*” OR“binge drinker*” OR “binge eat*” OR bipolar* OR “body dysmorphi*” OR “borderline personality disorder*” OR bulimi* OR Burnout OR “chronic stress” OR “combat disorder*” OR “compassion fatigue” OR “conduct disorder*” OR “conversion disorder*”OR Cope OR coping OR counseling OR “cyclothymic disorder*” OR “dependent personality disorder*” OR depressi* OR discriminat* OR distress OR “drug abuse” OR “drug craving” OR “drug dependence” OR “drug misuse” OR dysthymi* OR “eating disorder*” OR “EDNOS” OR “emotional distress” OR “emotional disturbance*” OR “emotional problem*” OR “emotionally unstable personality disorder* OR “fire setting behaviour*” OR “generalized anxiety disorder*” OR “Hazardous drink*” OR “histrionic personality disorder*” OR hypochondri* OR “impulse control” OR internali* OR “mental disease*” OR “mental disorder*” OR “mental distress” OR “mental health” OR “mental illness*” OR “mental stress” OR “Mood disorder*” OR “multiple drug abuse” OR “narcissistic personality disorder*” OR “narcotic analgesic agent” OR “narcotic dependence” OR “neurotic disorder*” OR“obsessive‐compulsive disorder*” OR “obsessive‐compulsive personality disorder*” OR “OCD” OR “OSFED” OR “panic disorder*” OR paranoi* OR “PCP” OR “personality disorder*” OR “phobic disorder*” OR “post‐traumatic” OR “post‐traumatic stress disorder*” OR Posttraumatic OR “prescription drug abuse” OR Psychiatric OR psychological OR psychosomat* OR psychotherapy OR “PTSD” OR purge* OR purging OR reject* OR “risky drink*” OR “schizoid personality disorder*” OR schizophren* OR “schizotypal personality disorder*” OR “sleep wake disorder*” OR “social phobia” OR Somatization OR somatoform OR stimulant* OR stress OR “substance abuse” OR “substance misuse” OR “Substance‐Related Disorder*” OR “trauma and stressor related disorder*” OR Trichotillomania OR health OR “Physical Health” OR “cardiovascular health” OR disease OR Illness OR “respiratory health” OR “brain health” OR “chronic disease” OR “Chronic illness”	AND	Ireland OR Irish OR “Republic of Ireland” OR Galway OR Dublin OR Belfast OR Cork OR Limerick

**FIGURE 1:**
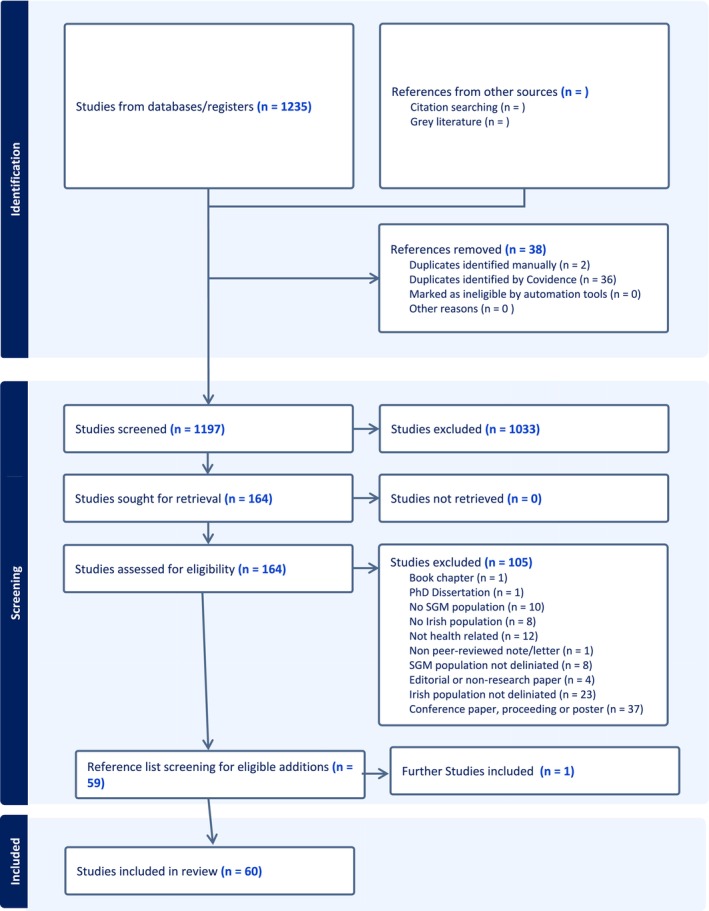
PRISMA ScR flowchart for scoping review (Page et al. 2021).

Once imported into Covidence, 38 additional duplicate records were identified and removed. This resulted in 1197 unique records that were screened for possible inclusion. All authors, apart from the specialist librarian, were involved in the screening process. At least two authors independently screened the title and abstract of each article. A total of 1033 articles were removed during this stage, leaving 164 for full text review. Two authors reviewed each full‐text article. At this stage, 105 studies were excluded for numerous reasons outlined in the Prisma flowchart (Figure 1). Following exclusions, reference lists were reviewed to ensure eligible articles were not missed, which led to the inclusion of one additional paper.

When discrepancies between authors arose at any stage, we consulted a third author to resolve the disagreement through a consensus‐based process. A total of 60 studies were included in the final review. The PRISMA ScR flowchart is seen below.

### 2.3 Data Extraction

Data were extracted and entered into a data extraction form constructed in Microsoft Excel (Supplementary File S1). The data extraction form was reviewed by authors independently to assess the form's suitability. The records were then divided among three authors for extraction. As data extraction congruency was almost 100%, discussions following the initial extraction centred on clarifications rather than revisions.

The lead author reviewed all extracted data for consistency in how the data were recorded and edited the extracted information for consistency in presentation. Extracted information included type of data (primary/secondary), study design, topic of the study, study location(s), aims of the study, sample size, SGM population group(s), inclusion of a heterosexual comparison group, racial/ethnic make‐up, age of the study sample, method of participant recruitment, primary independent variables/predictors, study outcomes, mediators/moderators, key findings and major limitations.

Terminology and acronyms used in the review to refer to sexual and gender minority populations (e.g., LGBT, LGBT+, LGBTQ+, SGM) reflect the terminology employed by the authors of the included studies. In our synthesis and analysis, we use the acronym SGM as an umbrella term to encompass the diversity of identities represented across the studies.

## 3 Results

### 3.1 Characteristics of Included Studies

Most included studies reported findings of primary research (*n* = 49), while a number of studies analysed existing clinical data (*n* = 8). One study used data from a large‐scale survey (Lochlainn et al. 2017) and another combined data from a cross‐sectional survey with existing clinical data (Moloney et al. 2021).

Most papers reported quantitative data (*n* = 37), while 19 reported qualitative data and four used mixed methods. The most common method of data collection was a survey (*n* = 33), 19 collected data using qualitative interviews and 6 conducted retrospective chart reviews. One study used a world café focus group approach and one clinical study collected clinical sample data prospectively.

The majority of studies focused solely on gay, bisexual and men who have sex with men (gbMSM) (*n* = 26), nine studies focused specifically on transgender and gender‐diverse populations, and only one study focused on bisexual people or lesbian women, respectively. The remainder of the studies (*n* = 22) included more than one SGM group. Most focused solely on adults (*n* = 44), a small number of studies focused only on adolescents (*n* = 4), with the remainder including both adolescents and adults (*n* = 11).

Across studies, detailed demographic information beyond age and sexual and/or gender identity was limited or inconsistently reported. This was particularly obvious regarding ethnicity, disability and socioeconomic status, limiting the ability to interpret how intersecting forms of marginalisation may intersect with health outcomes and experiences within SGM populations.

### 3.2 Mental Health

The greatest number of studies (*n* = 20) focused on mental health and included a range of topics such as sexual and gender identity differences in psychological distress (e.g., depression, anxiety), self‐harm and suicidality. Studies also examined factors that contribute to mental health disparities among SGM people such as discrimination and minority stress, as well as barriers to accessing mental health services. A summary of mental health articles can be found in Table 2.

**TABLE 2:** Summary of mental health articles.

Authors	Study design	Aim of study	SGM population	Key findings	
Bryan and Mayock (2017)	Mixed methods	To ascertain the prevalence of self‐harm and suicidality among LGBT‐identified persons living in Ireland and to identify factors and experiences that heighten vulnerability to psychological and suicidal distress.	LGBQ+	Prevalence of suicidal thoughts and actions: Majority of respondents never (42%) or only rarely (32%) considered suicide. Among younger participants (under 25), two‐thirds never seriously considered suicide in the past year, suggesting most are not at risk. High Lifetime Prevalence of Suicide attempts: 18% of the online sample and one‐third of the qualitative sample reported attempting suicide at least once. 14% considered suicide in the past 12 months, with one‐fifth of those making a plan and attempting suicide. 3. Complex Meanings of Suicidality: Analysis revealed diverse and complex meanings ascribed to suicidal feelings and actions, not always directly related to LGBT identification. 4. Challenging the “Suicide Consensus”: The study questions the widely accepted belief that LGBT youth are universally at elevated risk for suicidality and mental health issues. 5. Diverse Experiences: Many LGBT individuals reported that their mental health issues were not solely or primarily related to their sexual orientation or gender identity.	
Connolly and Lynch (2016)	Qualitative	To explore how the sexual orientation of gay men creates inequalities in the type of care delivered to them in the Irish health care system.	Gay men	Being gay in the Republic of Ireland: Persistent cultural challenges and homophobia despite legal progress. Being gay: Attitudes of the Catholic Church: The Church's negative views perpetuate stigmatisation and marginalisation. 3. Being gay: As a positive experience: Legal protections and social acceptance have improved some gay men's lives. 4. Being gay: As a negative experience: Societal stigma and internalised homophobia negatively impact mental health. 5. Coming out: Process and experience: Coming out is a complex, ongoing process that can be both liberating and stressful. 6. Coming out to health care professionals: Fear of stigma leads many gay men to hide their sexual orientation from healthcare providers.	
Cotter et al. (2014)	Cross‐sectional survey	To examine the association between sexual orientation concerns, mental health difficulties, and suicidal and risky behaviours among Irish adolescents.	LGB	Increased Victimisation: Adolescents with sexual orientation concerns faced significantly higher levels of victimisation. They were five times more likely to have been physically assaulted (40% vs. 8%) and one in six had been sexually assaulted (16% vs. 1%). There were stronger associations with substance misuse among these adolescents. One fifth of them drank alcohol frequently compared to 1% of their peers, and 75% were smokers compared to less than 20% of their classmates. Additionally, 41% had used hash or marijuana compared to 2% of their peers. 3. Peer Relationships: These adolescents did not significantly differ from their classmates in terms of getting along with peers or feeling liked and wanted in a group. 4. Romantic Relationships and Sexual Activity: They were more likely to have had a steady romantic relationship (43% vs. 11%) and experienced break‐ups (72% vs. 30%). A striking 90% reported having had sex compared to just 4% of their peers. Condom use was notably low among this group. 5. Parental Attention: They reported a lack of parental attention, involvement, and supervision. 57% said their parents rarely or never knew what they were doing in their spare time compared to 15% of their peers. 6. Mental Health and Well‐being: Adolescents with sexual orientation concerns exhibited higher levels of depressive symptoms, anxiety symptoms, emotional and behavioural problems, suicidal behaviour, and lower levels of well‐being. The most significant difference was in attempted suicide rates, with 29% of these students having attempted suicide compared to 2% of their peers.	
Cullinan et al. 2024	Cross‐sectional survey	To examine levels of psychological distress among higher education students in Ireland and explore variations across different personal, higher education, and socioeconomic characteristics.	LGBTQ+	High Prevalence of Psychological Distress: Significant proportions of students experienced mild to severe levels of depression, anxiety, and stress, highlighting a widespread mental health issue in higher education. Variations Across Demographic Groups: Higher distress levels were found among transgender, female, and LGBTQ+ students, undergraduates, and those from lower socioeconomic backgrounds or facing financial difficulties. 3. Institutional Differences: Students attending Institutes of Technology reported higher levels of severe psychological distress compared to those at universities, indicating the need for tailored mental health support.	
de Vries et al. (2023)	Cross‐sectional survey	To explore mental distress among transgender people in Ireland, using minority stress and cognitive dissonance theory.	LGBTQ+	Higher levels of mental distress, self‐harm, suicidal ideation, and lower self‐esteem among transgender participants compared to LGB groups and the general population. 53% of variance in mental distress could be predicted by reduced self‐esteem, experiences of harassment, and not belonging in school. 3. Mental distress was highest among younger participants, those not out, those who had self‐harmed, and those using avoidant coping.	
Higgins, Doyle, et al. (2016)	Qualitative	To explore resilience processes among older lesbian, gay, bisexual and transgender adults.	LGBT	Participants described multiple pathways to resilience, highlighting a range of processes that contributed to the development of resilient personality traits, such as courage, strength, a positive sense of self, and an optimistic outlook on life. They identified nine specific processes that enhanced their resilience, including: ‘Making a decision to accept oneself and not be defined by LGBT identity’; ‘Acquiring an empowering perspective’; ‘Learning to let go and moving on’; ‘Leaving oppressive social environments’; ‘Experiencing affirming relationships with family and others’; ‘Accessing formal supports’; and ‘Maintaining connections with LGBT people’.	
Higgins et al. (2021)	Cross‐sectional survey	To explore the barriers to accessing mental health services in the Republic of Ireland from the perspectives of young LGBT + people	LGBTQ+	Most participants (80.7%) reported barriers. NEED: Documented need: moderate levels of Depression, moderate Anxiety and mild Stress scores (DASS) were 16.6% (SD = 12.6), 13.3 (SD = 11.0) and 17.1 (SD = 10.9). In addition, 53.6% (*n* = 398) reported a history of self‐harm, just over two‐thirds had suicidal thoughts in their lifetime (69.4%, *n* = 507), and just over one‐quarter had attempted to take their own life (28.2%, *n* = 206). Barriers identified from the six items listed included the following: fear of the stigma of being labelled (38.9%, *n* = 414); prohibitive cost of private mental health services (37.2%, *n* = 396); a belief that services could not help them (25.5%, *n* = 271); and that services were not LGBTI + friendly (18.3%, *n* = 195). A further 17.0% (*n* = 180) reported knowing someone who had a bad experience of mental health services, while 15.8% (*n* = 168) had a bad experience themselves. Several additional barriers to accessing mental health services were identified across three levels: individua (e.g., beliefs about severity of need, lack of self‐confidence)l; sociocultural (e.g., stigma, lack of support from family); and mental health system (issues with accessibilty/availability, previous experiences, lack of provider cultual competence).	
Horgan et al. (2018)	Cross‐sectional survey	Describe, among first year undergraduate studetns, the prevalence of depressive symptoms, of suicidal ideation, characteristics of the sample. Explore the relationship between the participants' characteristics (e.g., age, gender, place of residence, relationship status, sexuality, parental relationship, alcohol use) and depressive symptoms and suicidal ideation.	LGB	A lower of percentage of heterosexual students experienced suicidal ideation compared to students with other sexualities (29.6%, 35.9%). Depression was not significantly different by sexual identity in the mutlivariate model.	
Költő et al. (2021)	Cross‐sectional survey	First. explore the prevelance of psychosocial phenomena including perceived discrimination and indicators included in Better Outcomes, Brighter Futures (BOBF) framework Outcome 5: “Connected, respected, and contributing to their own world” Second, compare indicators between sexual minority (same and both sex attracted) compared to non‐minority (opposite‐sex attracted and non attracted) peers.	LGB	Girls were more likely than boys to report discrimination based on gender and age. Frequency of positive answers ranged from 67% (feeling comfortable with friends) to 12% (being involved in volunteer work). Sexual minority youth were more likely to feel discriminated based on sexual orientation, age, and gender. Both‐gender attracted youth were less likely than the other groups to report positive outcomes. Same‐gender attracted youth were twice as likely as non‐minority youth to volunteer.	
Költő et al. (2023)	Cross‐sectional survey.	Examine perceived discrimination based on various grounds in four minority groups of schoolchildren in Ireland: sexual minority youth, youth living with a disability or chronic condition, immigrant youth, and youth belonging to the Traveller community.	LGB	Minority groups were significantly more likely than their matched non‐minority counterparts to report discrimination, not only on the basis of their minority status but also on other grounds. Large effect sizes were observed for sexual minority and Traveller adolescents. Compared their matched groups, sexual minority adolescents reported significantly higher rates of discrimination based on their gender, age, religion, and “other”	
Losty and O'Connor (2018)	Qualitative	Explore the psychological realities ofindividuals who self‐identified as having a non‐binary gender.	Trans	Three core themes emerged from the data: ‘A developing gender identity’ (development and meaning of identity), ‘Correct and incorrect language’ (relationship with referred pronouns and gender lables, being misgendered), and ‘Being seen and unseen’ (experiences of being validated/invalidated, seen/unseen). Invalidation was linked to varied levels of distress and, in some cases, poor mental health.	
Mahon, Fitzgerald, O'Reilly, and Dooley (2023)	Cross‐sectional survey	This study examined risky health behaviours, adverse health outcomes, and their overlap across mental, physical and sexual domains, in lesbian, gay, bisexual, questioning (LGBQ) and heterosexual third‐level students in Ireland	LGB	LGBQ students (both male and female) were more likely to exhibit a greater number of risky mental and sexual health behaviours and outcomes. Sexual, physical and mental health behaviours and outcomes overlapped to a greater extent in LGBQ versus heterosexual students.	
Mahon, Fitzgerald, O'Reilly, McDermott, et al. (2023)	Cross‐sectional survey	compare body esteem, body satisfaction, body change behaviours and risk/protective factors across sexual orientations.	LGBQ	Heterosexual men exhibited higher body esteem and body satisfaction than sexual minority men. Bisexual women demonstrated the lowest body esteem, while pansexual women exhibited lower body satisfaction versus heterosexual, lesbian and questioning women. Body change behaviours did not differ among women, but weight loss attempts were elevated in gay and bisexual men. Comfort with sexuality, resilience and social support were positively associated with body esteem. Risk and protective factors for body esteem varied by sexual orientation	
Mahon et al. (2021)	Cross‐sectional survey	To identify f determinants of social anxiety among sexual minority individuals	LGB	Discrimination and intraminority stress lead to social anxiety in sexual minority individuals via: Increased rejection sensitivity. Reduced sense of coherence. For sexual minority men: Intraminority stress also leads to social anxiety through increased concealment behaviour. For sexual minority women: Discrimination leads to social anxiety through: Increased minority stress (e.g., concealment behaviour, internalised homonegativity). Decreased LGBTQ community connectedness.	
McCann and Brown (2019)	Qualitative study	Examine the experiences of people who identify as LGBT+ in relation to their distinct psychosocial support needs	LGBTQ+	Significant challenges in accessing appropriate healthcare services tailored to LGBT+ needs. Difficulty navigating and accessing limited publicly funded services. Negative experiences with some practitioners' approaches and attitudes. Participants emphasised the need for better education, training, and competency development among healthcare providers.	
McCann and Brown (2020a)	Qualitative	To examine the views and experiences of bisexuals in relation to their distinct psychosocial support needs	Bisexual	Bisexuals experience poorer mental health when compared to other sexually diverse groups, such as lesbians and gay men. Negative expereince of disclosure to HCPs. Participants found it difficult to situate themselves in either heterosexual or lesbian, gay and transgender communities. Furthermore, there was an apparent lack of social support networks specific to bisexuals	
McCann and Sharek (2014a)	Qualitative	To examine the experiences of LGBT people concerning mental health services in Ireland.	LGBT	Participants expressed a strong desire for a review of existing services to better address the needs of LGBT individuals. They emphasised the importance of improving access to care, ensuring practitioners are knowledgeable and responsible, and offering a range of affordable therapeutic options, including accessible talking therapies. ong waiting lists and healthcare costs were burdensome for some. There was a strong desire for services that treat people with dignity and respect. Ideas to combat stigma and discrimination included public awareness campaigns. Participants called for better education for mental health practitioners on LGBT issues, suggesting workplace equality training and the development of good practice guidelines. The need for more support and psychoeducation for “silent carers” and significant others was emphasised. The impact of minority stress on young people and associated suicide risks was noted, along with the importance of educating young people about LGBT issues in schools.	
McCann and Sharek (2014b)	Mixed methods	To explore LGBT people's experiences of mental health service provision in Ireland	LGBT	A high number of respondents had mental health diagnoses, mainly treated with psychotropic medication, despite recommendations for talking therapies, which were rarely offered. Two‐thirds felt mental health services couldn't meet LGBT needs, and one‐third couldn't discuss LGBT issues with providers. Many experienced heterosexist attitudes, with staff assuming heterosexuality and not acknowledging LGBT partners. Staff were often seen as lacking knowledge of LGBT‐specific issues, highlighting the need for better education and training in compassionate, recovery‐oriented care. Transgender individuals faced significant prejudice and discrimination, emphasising the need for rights‐based, inclusive services. There is an urgent need to train mental health nurses to work effectively with LGBT individuals and their families, following interNational–ROI guidelines on human rights and diversity.	
McLafferty et al. (2017)	Cross sectional Survey (part of longitudinal study)	Te explore prevalence of mental health behavioural problems and treatment seeking among students	LGBTQ+	SGM specific: nearly three and a half times more likely to have a MDE, nearly four and a half times more likely to have GAD and five times more likely to have any lifetime disorder.	
Travers et al. (2020)	Cross sectional survey	To assess whether LGB status was associated with more trauma exposure and poorer mental health, and whether social support mediated these associations.	LGB	LGB status was significantly associated with increased trauma exposure and with symptoms of PTSD, depression and anxiety, but not with problematic alcohol use. These associations were mediated by social support from family only	

#### 3.2.1 Mental Health Outcomes Among SGM Populations in Ireland

Health research consistently shows significantly elevated mental health risks among SGM individuals in Ireland, especially adolescents and young adults. Across numerous studies, higher rates of depression, anxiety, suicidal ideation and self‐harm were reported compared to cisgender heterosexual peers. Nationally representative data (Cullinan et al. 2024; Higgins et al. 2021; Mahon, Fitzgerald, O'Reilly, and Dooley 2023) consistently show greater psychological distress, with transgender people reporting the most severe symptoms. Cotter et al. (2014) and McLafferty et al. (2017) also report elevated self‐harm and suicidality among SGM youth. Even where depression rates were similar (Horgan et al. 2018), suicidal ideation remained higher among sexual minority youth. Qualitative findings add context: Bryan and Mayock (2017) show distress is shaped more by intersecting life challenges than identity alone.

#### 3.2.2 Drivers of Mental Health Inequities

SGM mental health disparities are rooted in structural and interpersonal factors. Discrimination is a key driver, with Költő et al. (2021, 2023) and de Vries et al. (2023) highlighting the effects of identity‐based harassment, concealment and lack of belonging—particularly in school settings. Internalised stigma and community disconnection also contribute. Mahon et al. (2021) link internalised homonegativity and low SGM connectedness to anxiety, while Travers et al. (2020) link trauma exposure and weak social support to poor mental health among LGB university students. Mahon, Fitzgerald, O'Reilly, McDermott, et al. (2023) found bisexual and pansexual women reported lower body esteem and higher dissatisfaction, closely tied to comfort with sexuality and resilience. These findings point to the need to explore psychosocial dimensions like body image, self‐concept and affirmation in SGM mental health research.

#### 3.2.3 Mental Health Services: Access, Use and Acceptability

Despite these disparities, access to affirming mental health services remains limited. Structural and interpersonal barriers persist, including stigma, lack of provider competence and affordability. Higgins et al. (2021) found over 80% of LGBT+ youth faced barriers to care, such as fear of discrimination and concerns about inclusivity. McCann and Sharek (2014b) noted a tendency for clinicians to default to medication rather than offer talk‐based therapies. Qualitative studies (McCann and Sharek 2014a; McCann and Brown 2019) describe participants being dismissed, misgendered or made invisible, leading to delays, mistrust and heightened distress.

#### 3.2.4 Diverse Experiences Within the SGM Community

Disaggregated studies reveal varied mental health experiences across SGM subgroups. Bisexual individuals often feel excluded from both heterosexual and LGBT+ spaces. McCann and Brown (2020a) found that bisexual respondents struggled with identity disclosure and lacked support, leading to isolation and distress. Lesbian participants in McCann and Brown (2020b) expressed frustration with over‐reliance on pharmacological treatment and poor psychotherapy access. Non‐binary individuals also face distinct challenges. Losty and O'Connor (2018) describe how misgendering and erasure cause psychological harm and reduce engagement with care. Connolly and Lynch (2015) found that cultural and religious norms led some gay men to conceal their identities in clinical settings, exacerbating mental health difficulties.

### 3.3 Sexual Health

The second largest number of studies (*n* = 17) focused on sexual health, reflecting significant research on HIV and STI prevalence, prevention strategies, vaccination acceptability and broader sexual health behaviours among gbMSM. These studies address key topics such as demographic characteristics associated with HIV risk, the prevalence of various STIs and the impact of PrEP on reducing HIV transmission. Additionally, these studies explore factors influencing vaccination uptake, risky sexual behaviours and gaps in sexual health knowledge, as well as the stigma and disclosure challenges faced by HIV‐positive gbMSM. A summary of sexual health articles is found in Table 3.

**TABLE 3:** Summary of sexual health articles.

Authors	Study design	Aim of study	SGM population	Key findings
Carey et al. (2021)	Cross‐sectional survey	To identify factors associated with lower knowledge of HIV and STI transmission, testing, and treatment among MSM	gbMSM	36% had lower knowledge Younger MSM (18–24) had lower knowledge (aOR 1.98) 3. MSM with less than a degree had lower knowledge (aOR 1.58) 4. MSM living outside Dublin had lower knowledge (aOR 1.21) 5. MSM born in Ireland (vs outside) had lower knowledge (aOR 1.62) 6. MSM out to few or no people had lower knowledge (aOR 1.36 to 1.69) 7. MSM who never tested for HIV had lower knowledge (aOR 2.32) 8. Non‐engagement with Man2Man website associated with lower knowledge (aOR 1.81)
Cox et al. (2017)	Cross‐sectional survey	To assess the prevalence and bacterial load of *Gardnerella vaginalis* and Mollicutes in rectal swabs from men who have sex with men (MSM) and explore potential bacterial synergies.	gbMSM	High Prevalence of *G. vaginalis* : *Gardnerella vaginalis* was found in 83.2% of rectal swabs from MSM, indicating common presence or repeated exposure. Synergistic Co‐Infections: Co‐infections of *G. vaginalis* and *M. hominis* showed higher bacterial loads, suggesting significant pathogenic interactions. 3. Need for Routine Screening: The study emphasises the importance of routine screening for a broader range of bacteria in MSM to better manage and prevent infections.
Dolphin et al. (2018)	Cross‐sectional survey	To profile risky sex behaviours among college students using a range of psychosocial correlates.	LGBTQ+	High Prevalence of Risky Behaviours: 75% sexually active; 27.2% reported early sexual initiation, 29.5% had 5+ lifetime sexual partners, 12.1% had 2+ partners in the past 3 months. Predictors of Risky Behaviours: Significant predictors included demographic factors (undergraduate status, living arrangement), substance use, mental well‐being (anger, depression), and personal resources (self‐esteem, coping). Differences by Gender and Sexual Orientation: Differences in risky behaviours were noted between males and females, and among heterosexual, gay/lesbian, and bisexual students.
Keaveney et al. (2014)	Cross sectional survey & clinical samples	This study (A) investigated the prevalence of STIs in asymptomatic HIV‐infected men who have sex with men (MSM) attending a clinic for routine HIV care in the largest HIV‐centre in Ireland and (B) implemented a self‐screening option.	gbMSM	Part a 16% of HIV‐infected MSM screened were diagnosed with a STI. Thirty‐eight per cent reported always using condoms while 4% reported never using condoms, 46% used condoms inconsistently and 10% reported no sexual contacts in the preceding 12 months. For part B of the study, A self‐performed rectal swab was collected by 92% of patients indicating a high acceptability.
Kerr et al. (2022)	Retrospective chart review	Describes, in the context of changing patient demographics, the seroprevalence of vaccine preventable viral infections among attendees of the largest centre for HIV positive patients in Ireland	gbMSM	Comparing those who attended from 2014 onward compared to those who attended between 1987 and 2013: There was a significant increase whose aquisition risk was identified as MSM (68 vs. 36%, *p* < 0.001), but a decrease in those whose acquisition risk was heterosexual (25% vs. 42%, *p* < 0.001) and PWID (5 vs. 20%). The identified mode of HIV acquisition for almost half of the HIV positive cohort is MSM, followed by heterosexual acquisition in just over a third and injection drug use in less than a fifth of cases. MSM and PWID status was significantly associated with a decreased likelihood of mumps seronegativity, as was being of African origin.
Kerrigan and Green (2019)	Qualitative	Explores how previous exposure to religious homonegativity features in the sense‐making process following HIV diagnosis	Gay men	Two overaching themes: Negotiating authenticity in unsafe space, which relates to the experience of negotiating same‐sex attraction within religious environments, and Re‐emergence of religious shame in diagnosis, which relates to the way in which the men made sense of diagnosis from the position of having been exposed to religious homonegativity earlier in their lives.
Murphy et al. (2015)	Cross‐sectional survey	To investigate the relationship between HIV health optimism, HIV‐positive community attachment (HCA), gay community attachment (GCA) and HIV status disclosure to casual sex partners.	gbMSM	HIV Health Optimism is higher in those with lower disclosure. Community attachment between HIV community and wider gay community are somewhat oppositional.
Murphy et al. (2016)	Qualitative	To explore how non‐infectiousness due to antiretroviral therapy has affected HIV‐positive gay men's experience of serostatus disclosure to casual sex partners	gbMSM	Profound stigmatisation experienced by HIV‐positive gay men in Dublin's gay community, highlighting their social and sexual exclusion, blame, and non‐consensual disclosure of their HIV status. Self‐stigma signficiant. Lack of social support
Nic Lochlainn et al. (2017)	Cross‐sectional survey	To estimate the number likely to present for HIV PrEP in Ireland	gbMSM	Prediction of 9947 potential candidates for HIV PrEP
O'Connell et al. (2017)	Retrospective chart review	To determine trends in late presentation of HIV in Ireland	gbMSM	Reduction in late presentation among gbmsm
O'Connor et al. (2019)	Cross sectional survey	To explore factors associated with self‐reported STI diagnosis among MSM who were sexually active and had an STI test in the previous year	gbMSM	Having more than 2 casual sexual partners or condomless anal sex led to higher rates of STIs and use of geospatial apps was a factor in higher rates of sexual partners
O'Donnell et al. (2019)	Cross sectional survey	To improve understanding of HIV testing among MSM living in Ireland to inform prevention and testing initiatives	gbMSM	Inequality in testing, younger and lower educated gbMSM are less likely to have tested. Men outside of dublin less likely to have tested
O'Rourke et al. (2021)	Cross sectional cohort	To determine seroprevalence of human herpesvirus 8 in Ireland among blood donors, men who have sex with men, and heterosexual genitourinary medicine and infectious diseases clinic attendees	gbMSM	gbMSM more significantly higher levels of herpes, most prevalent in HIV positive group
Robinson et al. (2019)	Retrospective chart review	To examine factors associated with recent HIV infection.	gbMSM	gbMSM remain the largest group for new infections of HIV in Ireland
Sadlier et al. (2016)	Cross sectional survey	To examine HPV vaccine acceptability and factors influencing vaccine acceptability in MSM in Ireland	gbMSM	Cost a significant consideration for vaccine acceptability, general good acceptability. Poor knowledge overall
Sadlier et al. (2014)	Cohort study	Te explore prevalence of HPV among gbMSM	gbMSM	High prevalence of HPV 69% with DNA detected. HIV infection was positvely associated with higher rates
White et al. (2023)	Cross sectional survey	To determine the prevalence of self‐reported Hepatits vaccination in gbMSM in Ireland	gbMSM	Rates of hepatitis vaccination is high, factors negatiely impacting include living outside of Dublin, low education level and non‐engagement with sexual health services

#### 3.3.1 Epidemiological Evidence

Research shows that gbMSM in Ireland are disproportionately affected by HIV and STIs. Kerr et al. (2022), Robinson et al. (2019) and O'Connell et al. (2017) report a rise in HIV diagnoses among gbMSM, increasing from 36% to 68% over two decades. O'Connell et al. also found earlier diagnoses in gbMSM, suggesting stronger engagement with care. Robinson et al. used recent infection testing algorithms (RITA) to link new HIV cases with concurrent STIs, though data limitations affect surveillance.

STI co‐infection, especially among HIV‐positive gbMSM, is a recurring concern. Studies by Keaveney et al. (2014), Sadlier et al. (2014), Cox et al. (2017) and O'Rourke et al. (2021) found high rates of asymptomatic STIs, HPV (69%) and HHV‐8. Cox et al. reported 83% of rectal swabs tested positive for *Gardnerella vaginalis* in MSM, suggesting high carriage or repeated exposure.

#### 3.3.2 Prevention Practices and Biomedical Interventions

A shifting prevention landscape includes PrEP and vaccination. Lochlainn et al. (2017) estimated nearly 10,000 gbMSM in Ireland could benefit from PrEP. Keane et al. (2022) showed reduced HIV diagnoses after PrEP rollout but noted 32.6% of users contracted rectal STIs during COVID‐19, highlighting continued vulnerability.

Vaccination studies show progress and gaps. White et al. (2023) found high hepatitis A/B vaccination rates but noted disparities by geography, education and service engagement. Sadlier et al. (2016) reported HPV vaccine acceptability but low awareness and cost barriers.

#### 3.3.3 Knowledge, Risk and Behaviour

Sexual behaviour, knowledge and access shape risk. Dolphin et al. (2018) found early sexual debut, multiple partners, and substance use linked to poorer mental wellbeing among college students, with some identifying as LGB. Among sexually active gbMSM, O'Connor et al. (2019) found STI risk associated with condomless sex and casual partners, often facilitated through geosocial apps.

Knowledge gaps persist. Carey et al. (2021) and O'Donnell et al. (2019) found that younger, less‐educated gbMSM and those outside Dublin had lower HIV/STI knowledge and testing rates. Engagement with campaigns like Man2Man was associated with higher awareness.

#### 3.3.4 HIV Disclosure and Stigma

Despite medical advances, stigma around HIV remains pervasive. Murphy et al. (2015) found that higher treatment optimism reduced disclosure to casual partners. Attachment to the HIV‐positive community lowered disclosure, while attachment to the wider gay community increased it.

Murphy et al. (2016) noted that many HIV‐positive gay men faced internalised stigma, exclusion and non‐consensual status disclosure. Kerrigan and Green (2019) linked disclosure difficulties to religious shame and unaffirming environments, showing how stigma at the intersection of HIV and sexuality still shapes experience.

### 3.4 Transgender Health

Eight studies focus specifically on transgender health in Ireland and address four key themes, including experiences accessing gender‐affirming care, interactions with mental health providers, help‐seeking behaviours and psychological well‐being and the prevalence of autism traits. Collectively, these studies highlight the mental health challenges, systemic barriers and unique care needs of transgender individuals. A summary of transgender health articles is found in Table 4.

**TABLE 4:** Summary of transgender health articles.

Authors	Study design	Aim of study	SGM population	Key findings
Delaney and McCann (2021)	Qualitative	To explore the personal experiences of transgender people with Irish mental health services.	Trans	Affirmative experiences: Positive encounters with mental health services that validate and understand transgender identities. Non‐Affirmative Experiences: Negative encounters, including misgendering, dead‐naming, and lack of knowledge among clinicians, leading to distress and service attrition. 3. Clinician Relationship: Importance of trust, connection, and understanding in clinician‐service‐user relationships.
Howell and Maguire (2019)	Cross‐sectional survey	To compare transgender and cisgender participants in their likelihood to seek help for both physical and mental health conditions, and to explore whether this help‐seeking behaviour is predicted by sociodemographic and psychological variables.	Trans	Transgender participants were less likely to report seeking help for physical health problems than cisgender participants. No differences in seeking help for mental health; however transgender populations had significantly lower levels of optimism and self‐esteem compared to cisgender participants.
Judge et al. (2014)	Retrospective chart review	Update the characteristics of GD in Ireland, show the current prevalence of the condition	Trans	Diagnosis and referral of GD in Ireland is increasing. Specific findings: prevalence of GD in the Irish population was 1:10,154 male‐to‐female (MTF). and 1:27,668 female‐to‐male (FTM), similar to reported figures inWestern Europe. 159 of the patients were MTF and 59 were FTM, accounting for 72.9% and 27.1% of the cohort, respectively. The rate of referral increased, with 55 patients referred in 2013 versus 6 in 2005. Mean ages were 32.6 years (MTF) and 32.2 years (FTM). 22 of the patients were married and 41 had children, with 2 others having pregnant partners. 37.6% were referred by a psychologist, with the remainder evenly divided between GPs and psychiatric services. There were low rates of coexistent medical illness although psychiatric conditions were more prevalent, depression being a factor in 34.4% of patients. 5.9% of patients did not attend a mental health professional. 74.3% are currently on HT, and 9.17% have had gender reassignment surgery (GRS). Regret following hormonal or surgical treatment was in line with otherWestern European countries (1.83%).
Kearns et al. (2023)	Retrospective chart review	Describe demographics, referral pathwasy, and comorbidities of young adults presenting for gender services.	Trans	Incrasing referrals of AFAB over AMAD in Ireland. Transgender men represented 62.3% of the sample, transgender women 35.3%, and transmasculine/non‐binary individuals represented 2.4%. Over two‐thirds of participants were on gender affirming hormone therapy or GnRH antagonists and 16.1% had undergone surgical interventions. The median time from referral received to being seen at the clinic was 450 days (481 mean). Mental health comorbidities remain high with 49.1% of youth experiencing depression, a further 15.6% low mood and 26.3% anxiety.
Kearns et al. (2024)	Qualitative	Identify the common factors that help and hinder transgender and nonbinary youth accessing gender‐specific health care in Ireland and to identify how these factors may be perceived differently by young people seeking gender‐affirming care, their parents, and health‐care providers	Trans	Four themes were derived: (1) “Needing bricks to build” (structural factors); (2) “Enduring and convincing” (diagnostic factors); (3) “Being me, hiding me”; (personal factors); and (4) “It takes a tribe” (interpersonal factors). Each stakeholder group perceived different factors as help or hindrance in accessing care with varying intensities
Lehmann et al. (2020)	Retrospective chart review	Prevelance of autism among adolescents or adults seeking specialist gender services	Trans	Autism trait prevalence rates of 19.5% (AQ); 25.4% (RAADS‐14); and 35.8% (poor empathy traits). A combined measure comprising all three provided a prevalence of 17.2%. There were no mean differences in the scores between AMAB (assigned male at birth) individuals and AFAB (assigned female at birth) individuals. No differences by sexual orientation.
Lehmann et al. (2021)	Qualitative	Examined the experiences of treatment‐seeking adolescents and adults	Tras	Patients with gender dysphoria distrust clinical services and describe considerable anxiety in sustaining their impression management strategies to obtain treatment. An authentic presentation is regarded by some participants, especially non‐binary individuals, as inauthentic and emotionally difficult to maintain. Impression management strategies have partial success in accessing services. The presentation of “idealised” selves may result in unmet mental health needs of patients, and the receipt of interventions incongruent with their authentic selves.
McCann (2015)	Qualitative	To elicit the views and opinions of transgender people in relation to mental health concerns.	Trans	Trans people expereince frustrations involved in identifying, accessing and using thelimited available services as well as the challenges related to funding. Lack of support for families of trans people experience mental health difficulties. Continued experience of pathologising of trans identity

#### 3.4.1 Experiences Accessing Gender‐Affirming Care

Research shows that transgender healthcare in Ireland is marked by long wait times, rising demand and systemic barriers to gender‐affirming care. While not formally longitudinal, Judge et al. (2014) and Kearns et al. (2023) together trace this growing pressure. Judge et al. reported that over a third of patients at a specialist gender clinic had depression and most were on hormone therapy, with referrals already increasing—a trend Kearns et al. confirmed with a 412% rise in new referrals between 2013 and 2020.

Access is both a logistical and emotional challenge. Qualitative studies by Lehmann et al. (2021) and Kearns et al. (2024) show how individuals feel pressured to meet clinical expectations, often suppressing mental health histories or adopting binary narratives, particularly burdensome for non‐binary youth. Kearns et al. (2024) identify four themes: structural, diagnostic, personal and interpersonal, that shape how young people, parents and providers understand access, highlighting the emotional labour involved.

Medical chart reviews by Kearns et al. (2023) further show high mental health comorbidities among young adults, with nearly half reporting depression and over a quarter anxiety. Despite these challenges, most accessed hormone therapy, some had surgeries, yet waiting times often extended into years.

#### 3.4.2 Experiences With Mental Health Providers

Transgender individuals' experiences with mental health providers reveal both affirming and non‐affirming interactions. Delaney and McCann (2021) conducted a phenomenological interview study involving four transgender individuals, which highlighted both positive and negative encounters. Affirming interactions, characterised by validation and understanding of transgender identities, were associated with improved outcomes. However, negative experiences, such as misgendering, dead‐naming (using a person's birth name rather than their chosen name) and a lack of clinician knowledge, often led to distress, health‐seeking avoidance and service attrition. McCann (2015) echoed these findings in their qualitative study, which involved interviews with four transgender participants aged 28–54, recruited through LGBT and mental health organisations. Participants reported frustration with limited service availability, funding challenges and continued pathologising of transgender identities. Both studies underscore the role of clinician knowledge and sensitivity in delivering effective mental health support for transgender individuals, with non‐affirming interactions contributing to significant barriers in accessing care.

#### 3.4.3 Help‐Seeking Behaviours and Psychological Well‐Being

The challenges of accessing gender‐affirming care intersect closely with broader help‐seeking behaviours and psychological wellbeing among transgender individuals. Howell and Maguire (2019) found that while transgender and cisgender individuals report similar rates of seeking mental health support, transgender individuals are significantly less likely to seek help for physical health concerns. This reluctance was associated with lower self‐esteem and optimism, and likely reflects broader systemic mistrust, stigma and fear of negative experiences within medical settings. These findings echo the emotional labour described by Lehmann et al. (2021) and Kearns et al. (2024), where trans and non‐binary individuals feel compelled to conform to narrow clinical narratives to be seen as legitimate recipients of gender‐affirming care.

Help‐seeking, whether for general or gender‐specific health needs, is influenced by psychosocial factors and shaped by a broader context of marginalisation. The medical chart reviews conducted by Judge et al. (2014) and Kearns et al. (2023) document extensive mental health burdens among those pursuing gender‐affirming interventions, while also highlighting lengthy wait times and high demand for care. Taken together, these findings suggest that reluctance to seek care is not a reflection of individual avoidance, but rather a response to systemic structures that often fail to provide inclusive, affirming or accessible services.

#### 3.4.4 Autism Traits and Gender‐Affirming Care

Two in the review highlight the importance of recognising neurodiverse needs within transgender healthcare. Lehmann et al. (2020) found that around one in five adolescents and adults attending gender services displayed autism traits across standardised assessments. These traits were not associated with sex assigned at birth or sexual orientation, suggesting that neurodiversity is common across gender and sexual identities. Similarly, Kearns et al. (2023) found that 11.3% of their clinical sample had a formal diagnosis of autism spectrum disorder (ASD), with an additional 2.9% showing clinical features consistent with ASD. The authors noted that their chart‐based method likely underestimates the true prevalence.

### 3.5 Substance Use

Six studies specifically examined issues related to substance use, all focused on gbMSM individuals. Three cross‐sectional surveys focused on prevalence and correlates of substance use. Five of the six studies included, or focused specifically, on the issue of chemsex, which refers to the sexualised use of certain substances such as methamphetamine, GHB/GBL, mephedrone, cocaine and ketamine in communities of gbMSM (Maxwell et al. 2019). A summary of substance use articles is found in Table 5.

**TABLE 5:** Summary of substance use articles.

Authors	Study design	Aim of study	SGM population	Key findings
Barrett et al. (2018)	Cross‐sectional survey	To measure the prevalence of recreational drug use among MSM in a National–ROI sample, and to identify sub‐groups of MSM who may benefit from targeted preventive interventions.	gbMSM	High Prevalence of Drug Use: 36% of MSM used recreational drugs, 33% used poppers, and 7% used chemsex drugs in the past year. Age and Drug Use: Younger MSM (median age 27) were more likely to use recreational drugs, while older MSM (median age 34) were more likely to use poppers. 3. HIV Status and Drug Use: Higher odds of drug use among HIV‐positive MSM: • Recreational drugs: AOR 2.27• Poppers: AOR 3.77 • Chemsex drugs: AOR 5.87 4. Geographical Variation: Higher prevalence of drug use among MSM living in Dublin. 5. Associated Behaviours: Clustering of risk behaviours; drug users were also more likely to binge drink and smoke.
Daly et al. (2021)	Cross‐sectional survey	To quantify the prevalence of potential Alcohol Use Disorder (AUD) among men who have sex with men (MSM) in Ireland and to identify the key demographic, behavioural, and psychosocial characteristics associated with potential AUD.	gbMSM	31% of MSM screened positive for AUD. Higher odds of AUD among MSM who were bisexual, native to Ireland, unemployed, used illicit drugs, reported anxiety/depression, or experienced homophobic abuse. 3. Students were less likely to screen positive for AUD.
Daly et al. (2023)	Cross‐sectional survey	To quantify the prevalence of recreational drug use (RDU) and sexualised drug use (SDU) among gbMSM in Ireland and to identify the factors associated with these drug use practices.	gbMSM	Among gbMSM without HIV, 40.9% engaged in RDU and 13.1% in SDU in the previous year. Among gbMSM with HIV, 51.8% engaged in RDU and 26.2% in SDU in the previous year. Increased odds of RDU among younger gbMSM, those residing in Dublin, and those engaging in CAI. Increased odds of SDU among those residing in Dublin, engaging in CAI, and those with a bacterial STI diagnosis.
Glynn et al. (2018)	Cross‐sectional survey	To assess the prevalence of chemsex, associated behaviours, and sexually transmitted infections (STIs) among MSM attending a sexual health clinic in Dublin.	gbMSM	27% of respondents reported engaging in chemsex within the previous 12 months. Chemsex was associated with having more sexual partners, more anal intercourse partners, and engaging in condomless anal intercourse. Those engaging in chemsex were more likely to have been treated for gonorrhoea in the past 12 months. One in four (25%) reported that chemsex was impacting negatively on their lives and almost one third (31%) reported that they would like help or advice about chemsex
Joyce et al. (2018)	Qualitative	Explore problematicGuse as experienced by people who had presented for treatment	gbMSM	Three superordinate themes reflecting the participants chronological development of problematic ‘G’ use and early recovery were fully developed and presented—(1) early use: BI was part of that gang that took G; (2) daily use: BIt's like insidious; it just weaves into your everyday life; and (3) early recovery: my priorities are changing.
Van Hout et al. (2019)	Qualitative	To explore the experience of chemsex	gbMSM	Chemsex seen as changing the gay social networks of Dublin. Reflection on the interplay between sex and drug dependence. Studey reported on harm reduction activities engaged in

#### 3.5.1 Prevalence Studies

Two studies examined recreational drug use and chemsex among gbMSM in Ireland using cross‐sectional internet surveys (Barrett et al. 2018; Daly et al. 2023). Daly et al., using 2017 EMIS data, found higher rates of recreational (51.8%) and sexualised drug use (26.2%) among gbMSM with HIV compared to those without HIV (40.9% and 13.1%). Barrett et al. reported 36% used recreational drugs, 33% used poppers and 7% used chemsex drugs in the past year. Both studies found higher drug use among younger gbMSM and those living in Dublin. Older gbMSM were more likely to use poppers.

Risk behaviours were also associated. Daly et al. found recreational and sexualised drug use was linked to condomless anal intercourse and STI diagnoses. Barrett et al. found gbMSM with HIV had significantly higher odds of using drugs and were more likely to binge drink and smoke.

Daly et al. (2021) also examined alcohol use disorder (AUD) among gbMSM using EMIS data. Thirty‐one percent screened positive for AUD, with higher rates among bisexual men, Irish‐born individuals, the unemployed and those using illicit drugs. Students were less likely to report AUD. AUD was also associated with homophobic abuse and anxiety/depression.

#### 3.5.2 Chemsex

One of the three studies focused explicitly on chemsex (Glynn et al. 2018) used cross‐sectional survey data from 510 gbMSM (90.1% gay, 7.8% bisexual) attending a sexual health clinic in Dublin to assess the prevalence of chemsex, as well as associated risk behaviours and sexually transmitted infections (STIs). The authors found that 27% of the sample had engaged in chemsex within the past 12 months. Chemsex was associated with having more sexual partners, engaging in more anal intercourse, and condomless anal intercourse, and MSM who engaged in chemsex were also more likely to have been treated for gonorrhoea in the past year. Additionally, one in four participants reported that chemsex was negatively impacting their lives, and almost one‐third wanted help or advice about chemsex.

Complementing this epidemiological evidence, two qualitative studies examined experiences of drug use and chemsex, both of which highlighted the importance of social networks and community context. Van Hout et al. (2019) conducted in‐depth interviews with10 gbMSM engaged in chemsex in Dublin, finding that chemsex reshaped social networks within the gay community, blurring the lines between socialising and drug use, with harm reduction strategies often employed to mitigate risks. Joyce et al. (2018) explored problematic GHB/GBL use among individuals (five gay men and two women) who presented for treatment with a community drug team in Dublin. Three key themes regarding the development of problematic G use were identified: early use (‘part of the gang that took G’), daily use (’insidious, weaving into everyday life’), and early recovery (’my priorities are changing’). Some participants noted that, although chemsex was part of daily use among gay men, combining G and sex sometimes diminished as problematic drug use increased. The findings highlighted the role of social networks in the initiation and escalation of G use and the importance of connecting with help, finding new ways of connecting with community, re‐thinking priorities and examining the ambivalence associated with recovering from problematic use.

These studies point to chemsex as a phenomenon situated at the intersection of sexual health, community belonging and substance use.

### 3.6 Cancer

Three studies explored various aspects of cancer care among SGM populations, specifically focused on the experiences of gay men, transgender and non‐binary individuals. Across the studies, themes of identity erasure, inadequate cultural competence and the need for tailored support recur, with implications for both physical and psychosocial outcomes. A summary of cancer articles is found in Table 6.

**TABLE 6:** Summary of cancer articles.

Authors	Study design	Aim of study	SGM population	Key findings
McConkey and Holborn (2018)	Qualitative	To explore the experiences of Irish gay men of prostate cancer	Gay men	Overall negative impact of prostate cancer on the lives of gay men. The physical effects of cancer and its treatment can severely impact gay men's psychological, social well‐being, and overall quality of life. This distress is worsened by healthcare providers' lack of knowledge and communication skills regarding treatment decisions, gay men's sexual practices, and limited community‐based support from cancer charities and the gay community.
Moloney et al. (2021)	Retrospective review of medical records and cross‐sectional survey	To identify the areas of care that could be improved for cancer care of Trans and Non Binary people	Trans	Discrepency between affirmed name and gender pronouns between outpatient letters and health records. Some patients had hormones postponed during cancer treatment. Misgenering was reported in the survey, and this had a negative impact on mental health
Saab et al. (2023)	Qualitative study–World café focus group	To co‐design an inclusive community‐based campaign to promote testicular awareness	LGBTQ+	Social media an important method of education, offline communication can supplement this. Focus should be on challenging embarassment. Campaigns should be tailored to different groups

McConkey and Holborn (2018) conducted a phenomenological study with gay men on their experiences of prostate cancer. The study found that cancer and its treatment had a severe impact on psychological and social well‐being and quality of life. Participants reported distress over healthcare providers' lack of understanding of gay sexual practices and the absence of tailored support from both cancer charities and the gay community.

Similarly, Moloney et al. (2021) examined cancer care experiences but among five transgender and gender‐diverse individuals using a retrospective clinical review and cross‐sectional survey. The study found that mismatches between affirmed names/pronouns and medical records negatively affected mental health. Some participants also experienced interruptions in gender‐affirming hormone therapy during cancer treatment.

Using World Café methodology, Saab et al. (2023) co‐designed a testicular health awareness campaign with 17 SGM participants (mostly gay men). Social media was preferred for education, with offline options as secondary. The study emphasised addressing embarrassment around testicular health and tailoring messages for inclusivity and relevance across SGM groups.

Collectively, these studies reveal that cancer care systems in Ireland are not yet adequately prepared to meet the needs of SGM populations. Whether through gaps in provider knowledge, failures in record‐keeping or absence of affirming community supports, SGM individuals often face layered exclusions that compound the challenges of diagnosis and treatment.

### 3.7 Specific Healthcare Needs of Older People

Two papers reported on a mixed‐methods study exploring the healthcare experiences and concerns of older LGBT people in Ireland, involving participants aged 55–74, recruited via LGBT websites, older people's forums and national events. Sharek et al. (2015) presented quantitative findings from 144 survey respondents: only one in three believed healthcare professionals had sufficient LGBT knowledge, and just 43% felt respected as LGBT individuals. A quarter (26%) had concealed their identity due to fear of negative reactions.

Higgins, Sharek, and Glacken (2016) reported qualitative findings from 36 interviewees, most aged 55–59 (61%). The sample was 61% male, 31% female, 6% trans and 3% other. Participants described various pathways to resilience, including courage, optimism and self‐acceptance. Nine key processes were identified, such as refusing to be defined solely by LGBT identity, adopting empowering perspectives and learning to let go and move forward. A summary of articles on the healthcare needs of older people is found in Table 7.

**TABLE 7:** Summary of healthcare needs of older people papers.

Authors	Study design	Aim of study	SGM population	Key findings
Higgins, Doyle, et al. (2016)	Qualitative	To explore resilience processes among older lesbian, gay, bisexual and transgender adults.	LGBT	Participants described multiple pathways to resilience, highlighting a range of processes that contributed to the development of resilient personality traits, such as courage, strength, a positive sense of self, and an optimistic outlook on life. They identified nine specific processes that enhanced their resilience, including: ‘Making a decision to accept oneself and not be defined by LGBT identity’; ‘Acquiring an empowering perspective’; ‘Learning to let go and moving on’; ‘Leaving oppressive social environments’; ‘Experiencing affirming relationships with family and others’; ‘Accessing formal supports’; and ‘Maintaining connections with LGBT people’.
Sharek et al. (2015)	Cross sectional survey and qualitative	To explore experiences and preceptions of older LGBT people of healthcare servies in Ireland	LGBT	25% not out to healthcare practitioners. Experience of discrimination was not common but participants felt needs are not met

### 3.8 Happiness and Community Connection

A study by Ceatha (2016) and Ceatha et al. (2019) explored the concept of well‐being among LGBT people in Dublin through a qualitative study. The research involved 11 participants, including lesbian, gay, bisexual and transgender individuals, aged 22–56 years. The study used in‐depth interviews and focused on participants' understanding of wellness through engagement in interest‐sharing groups. Findings revealed that participants described ‘mastering wellness’ through active participation in interest groups, which helped boost self‐esteem and create a balance in their lives. Participants emphasised the therapeutic benefits of shared hobbies and interests, as well as the sense of community and support found within these groups.

Complementing these findings, de Vries et al. (2020) mixed methods study examining happiness among LGBTI individuals in Ireland highlighted that predictors of higher happiness included strong self‐esteem and being in a relationship. The sample included 2264 participants, predominantly gay men (38.6%) and lesbian women (26.5%), with ages ranging from 14–46+ years. The study measured happiness and life satisfaction using an 11‐point scale and explored self‐esteem, stress and coping strategies. Overall, the mean happiness rating among participants was 6.58 out of 10, lower than the general population in Ireland. Gay men and lesbian women reported higher happiness compared to bisexual, transgender, or intersex participants. Younger LGBTI participants (14–18 years) reported significantly lower happiness ratings. Experiencing LGBTI‐related violence was a key factor negatively impacting happiness.

While Ceatha et al. underscore the value of community and shared activity for fostering wellness, de Vries et al. point to the vulnerabilities experienced by younger and more marginalised subgroups within SGM communities. A summary of articles on happiness and community engagement is found in Table 8.

**TABLE 8:** Summary of happiness and community connection papers.

Authors	Study design	Aim of study	SGM population	Key findings
Ceatha (2016)	Qualitative	To explore LGBT people's understanding of their wellbeing through interest sharing	LGBTQ+	Mastering wellness: Participants described mastering wellness through active engagement in interest groups, emphasising personal agency and proactive approaches to mental and emotional health. Experiencing mental health: Participants felt comfortable discussing their mental health openly, attributing positive management to the support and connection within interest‐sharing groups. Broadening the Concept of Well‐being: Participants broadened well‐being to include the active pursuit of interests and hobbies, which they found therapeutic and beneficial for mental health. Building Self‐Esteem: Involvement in interest groups significantly boosted participants' self‐esteem, with positive reinforcement and skill development fostering a sense of achievement and self‐worth. Creating balance: Participants highlighted creating balance in their lives through engagement in interest groups, counteracting stressors and maintaining a healthy work‐life balance for mental well‐being.
Ceatha et al. (2019)	Qualitative	To explore the social meaning and significance of LGBT social and community through their sporting, creative or social pursuits.	LGBTQ+	Connecting through shared Interests: shared interests like food, hiking, and social activities helped create social networks and a sense of belonging within the LGBT community. Connecting through Skill: Activities like creative writing, choir, and sports allowed skill development and peer validation, enhancing participants' sense of competence and community contribution. Connecting through LGBT Identity: Shared interests facilitated integration into LGBT groups, helping participants merge their sexual orientation and gender identity with their interests. Connecting Socially: Social connections within LGBT groups provided alternatives to pub‐club scenes, offering belonging and recognition, especially during the coming‐out process. Connecting with LGBT Communities: Participants felt part of a wider, inclusive LGBT network, emphasising visibility and empowerment through involvement in diverse community groups.
de Vries et al. (2020)	Cross‐sectional survey and qualitative study	To explore factors that contribute to happiness among LGBTI individuals as part of a comprehensive study on mental health within the LGBTI community in Ireland.	LGBTQ+	Mean happiness rating was 6.58 out of 10, lower than the general population in Ireland. Gay men and lesbian women reported higher happiness than bisexual, transgender, or intersex participants. Younger LGBTI participants (14–18 years) had significantly lower happiness ratings compared to older age groups. Self‐esteem and being in a relationship were significant predictors of happiness. Experiencing LGBTI‐related violence negatively impacted happiness.

## 4 Discussion

This scoping review highlights a number of interrelated trends in SGM health research in Ireland. Across the studies, we found persistent mental and physical health disparities, systemic exclusion in healthcare and gaps in research inclusive of diverse SGM subgroups. For example, we found that a majority of studies focused on gbMSM and a lack of inclusion of other SGM groups, particularly intersex people and sexual minority women. These gaps limit the capacity of health entities and providers to respond to the full spectrum of LGBTQI health experiences and needs. We also found limited or inconsistent collection or incorporation of diverse demographics in analyses. Consequently, intersectionality and the effects of multiple marginalisation are inadequately explored, constraining the analytical depth of current research. Nevertheless, there is emerging evidence of the protective effects of community, resilience and peer support across diverse SGM populations.

### 4.1 Minority Stress as a Main Interpretive Lens

Numerous studies within this review specifically frame their understanding of SGM health disparities using the Minority Stress model (Brooks 1981; Meyer 2003). This model provides a framework for understanding how stressors, both external and internal, that are unique to SGM individuals accumulate and contribute to health disparities observed in SGM populations (Flentje et al. 2021; Frost et al. 2015; Hoy‐Ellis 2023; Hughes et al. 2023; Lick et al. 2013; Zeeman et al. 2019).

Meyer (2003, 2014) describes two types of minority stressors: distal and proximal. Distal stressors are external, objective experiences such as discrimination, harassment, social exclusion, or violence that SGM individuals experience because of their sexual orientation or gender identity. These stressors reflect societal bias and oppression. Within this review, McCann (2015) and Delaney and McCann (2021) highlight the discrimination faced by transgender individuals in healthcare settings, including misgendering and the lack of provider knowledge lack of provider knowledge, which fosters distrust, service avoidance and poor outcomes. Structural barriers to affirming care intensify personal experiences of stress, reinforcing cycles of exclusion and unmet needs.

Proximal stressors are internalised stress responses that arise due to the social context such as anticipating rejection due to past discrimination, leading to anxiety and hypervigilance. In this review, de Vries et al. (2023) and Mahon et al. (2021) apply the minority stress model to show how rejection sensitivity and lack of community connectedness compound mental health challenges. SGM youth facing bullying and exclusion experience higher rates of anxiety, depression and suicidal ideation, findings consistent with international evidence (Hoy‐Ellis 2023).

The minority stress model also highlights the role of protective factors, such as social support and coping strategies. Several studies in this review highlight the role of community in promoting wellbeing (Ceatha 2016; Ceatha et al. 2019; de Vries et al. 2020; Költő et al. 2021). Ceatha emphasised the importance of connectedness and group participation as resilience building, while De Vries et al. found strong self‐esteem, relationships and community involvement linked to happiness and life satisfaction.

A notable issue in the review is the absence of other theoretical frameworks to understand health inequities, suggesting a need for greater theoretical pluralism. Irish researchers might consider models that address limitations of the minority stress model, such as its underemphasis on positive and physical health outcomes (Vaughan and Rodriguez 2014; Lick et al. 2013), intraminority and intersectional stressors (Bowleg 2008; Pachankis et al. 2020), belonging to wider communities (Hoy‐Ellis 2023) and varied timescales of stress (Rivas‐Koehl et al. 2023). Some scholars argue for moving beyond minority stress entirely, proposing that a lack of social safety is a more accurate root cause of SGM health inequalities (Diamond and Alley 2022), whereas minority stress can risk individualising the effects of heteronormativity (Linander et al. 2024).

This highlights the value of ecological models that consider interactions between individual, community, institutional and structural factors (van der Star 2024), drawing on systems theory as in Brooks (1981) and Rich et al. (2020), given the complex, non‐linear nature of health inequalities (Moore et al. 2021). A more radical approach would involve developing theories not rooted in positivism, opening avenues to explore how power shapes health inequalities, how health discourses position SGM communities in Ireland, and how the absence of certain supports impacts wellbeing. Critical approaches also caution that disparity models may unintentionally reinforce stereotypes by comparing SGM communities to heteronormative ones (Frost 2017). For instance, minority stress‐based research often frames coping behaviours as risky (Hascher et al. 2023) or presents SGM individuals as inherently vulnerable, as in Bryan and Mayock (2017). This neglects the diversity of queer lives and how alternative ways of being can shape health in complex and potentially positive ways. To move beyond the limitations of an exclusively risk‐based model, researchers could draw on alternative or complementary frameworks that address health inequities in a more holistic way. One such example is the Health Equity Promotion Model (Fredriksen‐Goldsen et al. 2014), which was developed specifically for SGM populations and centres the role of historical, structural and social determinants of health. Importantly, it takes a strengths‐based approach, incorporating resilience, community engagement and life‐course considerations alongside risk. This model may be particularly useful in addressing the kinds of disparities observed in the reviewed literature, such as barriers to care, mental health burdens and social isolation, while also capturing protective factors like community support and identity affirmation.

### 4.2 Lack of Research With Groups Other Than gbMSM

Most sexual health studies reviewed, such as those by Kerr et al. (2022) and Keaveney et al. (2014), centre on HIV and STI prevalence, prevention and risk behaviours in gbMSM communities. While the need for HIV prevention and screening among this group is indisputable, the lack of attention to other sexual and gender minority groups reveals a broader issue of exclusion within SGM health research. This disparity in focus can reinforce the invisibility of other SGM individuals in health discourse and service provision.

For example, sexual minority women are grossly underrepresented in the reviewed literature, despite evidence from international research showing that lesbians and bisexual women often experience unique health risks, including lower rates of STI screening, higher rates of smoking and alcohol use, and increased risk of certain cancers (Hughes and Sommers 2015; Hughes et al. 2020b). Within this review there is only a single study specifically focused on sexual minority women. While this represents a significant gap in the Irish evidence base, similar patterns are evident internationally. In their systematic review of SGM women's health in the UK, Meads et al. (2019) highlight a twofold underrepresentation: not only is research on SGM populations limited overall, but studies that focus specifically on SGM women are even scarcer.

The complete absence of research on intersex individuals in this review further highlights the gaps in understanding the health of sexual and gender minority populations. While there is no formal census of the intersex population in Ireland, international research has generally estimated that 1.7%–4% of the population will have intersex variations (Jones 2018). The lack of research on intersex health is not unique to Ireland but reflects a widespread international gap in health research. Globally, intersex individuals are frequently excluded from health studies, surveillance systems and policy frameworks, resulting in a limited evidence base to inform appropriate care (Zeeman and Aranda 2020). This invisibility contributes to continued stigma, inadequate clinical guidance and a failure to address the distinct healthcare needs of intersex people.

This research imbalance has real‐world implications, particularly in the design of health interventions and services that often fail to consider the diversity within the SGM communities.

### 4.3 Lack of Sexual Orientation and Gender Identity Data in Irish Health Systems

A majority of the studies included in this review relied on convenience sampling and online surveys. While such methods are, of course, common and effective for reaching marginalised populations, they also highlight the absence of robust, population‐level SOGI data in Irish health systems and national prevalence studies. The absence of routine SOGI data collection in healthcare settings prevents a clear understanding of the health disparities faced by SGM individuals at both a clinical care level and also in health research (Gilmore et al. 2024; Hughes et al. 2023; Hughes et al. 2022). It also limits the ability to develop tailored health services and monitor outcomes over time. Even use of larger data‐sets such as the MSM Internet Survey used in Nic Lochlainn et al. (2017) and Carey et al. (2021), while effective in reaching specific populations, risks excluding SGM individuals who are not part of online communities or who face digital exclusion.

Moreover, the lack of SOGI data in national health databases mirrors broader structural issues within Irish health policy. Without data on the prevalence of SGM individuals across the population, as could be obtained through national census data, it is difficult to quantify the scale of health needs and allocate appropriate resources. The absence of census data on SGM people in Ireland also reflects a broader cultural reluctance to fully integrate sexual and gender minority issues into public policy. This lack of visibility perpetuates the underfunding and underdevelopment of SGM‐specific health services, particularly for groups like transgender and non‐binary individuals who often face the greatest barriers to accessing appropriate care.

### 4.4 Intersectionality and the Unique Challenges Faced by SGM Subgroups

As noted in the results section, there was limited information across the studies around wider demographic and intersectional characteristics such as race, ethnicity, social class, disability etc. which limit our understanding of how intersectional oppression might impact sexual and gender minorities in Ireland. The limited intersectional understanding of SGM health inequities is not unique to Ireland; globally, much health research still adopts a single‐axis lens that centres whiteness and overlooks overlapping identities (Bowleg et al. 2023). While international studies offer insights into multiple marginalisation, it is vital to consider identities and social conditions specific to Ireland.

One such group is Irish Travellers, an Indigenous ethnic minority with significant health disparities, including reduced life expectancy (Quilty and Kennedy 2024). These are likely exacerbated for LGBT Travellers, who experience compounded marginalisation (Sartori 2022).

Another neglected issue is Ireland's homelessness crisis. Since 2015, emergency accommodation use has more than doubled, with family homelessness rising over 300% (Allen et al. 2020; Department of Housing, Local Government and Heritage 2022; Long et al. 2019). International evidence shows SGM people are overrepresented among homeless populations due to rejection and lack of support (Fraser et al. 2019), a pattern mirrored in Ireland. Belong to Youth Services (2021) reports elevated homelessness risk among SGM youth, often linked to family conflict over identity.

Migrant racialisation is another key intersection absent in the reviewed literature. Ireland's Direct Provision system, known to harm mental health, presents distinct challenges for SGM asylum seekers, whose experiences remain largely undocumented (Noone et al. 2018).

In general, research suggests that those who experience multiple forms of marginalisation, such as SGM youth who are also from racial or socio‐economic minority groups, are more likely to experience poorer health outcomes (McDermott et al. 2021). The lack of intersectional analysis revealed in this review limits the ability of research to capture the full complexity of SGM individuals' experiences in Ireland and reinforces the invisibility of multiple marginalised individuals within the health system.

### 4.5 The Role of Community and Social Support in Mitigating Health Risks

Although many of the studies focused on specific health disparities or inequalities within the health system, findings from some studies suggest that fostering SGM community networks and peer support can mitigate some of the mental health challenges associated with minority stress (Költő et al. 2021; de Vries et al. 2020).

While evidently clinical mental health services need to be more accessible and inclusive, there should also be greater investment in community‐based support systems that are culturally competent and attuned to the specific needs of diverse SGM populations.

## 5 Limitations

This review provides a comprehensive overview of SGM health research in Ireland but has several noted limitations. In line with scoping review methodology, we did not assess the quality, risk of bias or other limitations within individual studies. Additionally, we made the decision to focus solely on research published in peer‐reviewed journals, excluding studies commissioned by community organisations or health agencies that have not been published in scholarly outlets. This exclusion may have resulted in the omission of valuable insights from non‐scholarly sources that could have enriched the review.

Organisations such as BeLonG To, TENI, LGBT Ireland and regional Pride networks have historically provided formal and informal support for SGM individuals across Ireland, particularly for youth and transgender people navigating exclusion from families, schools or healthcare. For example, research by Ceatha (2016) and Ceatha et al. 2019 in this review highlights how SGM individuals in Dublin described ‘mastering wellness’ through participation in interest‐sharing groups that fostered self‐esteem and belonging. These findings are supported by de Vries et al. (2020), who found that self‐esteem, community involvement and social relationships were key predictors of happiness among LGBTI participants in Ireland.

However, such supports are unevenly distributed. Rural SGM individuals, older people, ethnic minorities and those with limited access to digital or transport infrastructure may struggle to engage with community networks. While cities like Dublin and Galway have relatively well‐developed SGM infrastructure, other areas lack sufficient investment in affirming spaces and services. The historical evolution of Irish SGM civil society, from a focus on decriminalisation and marriage equality to current campaigns for trans healthcare and inclusion, has shaped which groups are most visible and supported. Some subgroups, particularly intersex people and ethnic minority SGM communities, remain underrepresented in both civil society initiatives and research.

While intersectionality was identified as a key gap in the literature, this review is also limited by the paucity of data disaggregated by ethnicity, class, disability, migration status, or other social categories. SGM communities are not a monolith, and the lack of intersectional approaches in the included studies means many voices, particularly those at the intersections of multiple forms of marginalisation, are not adequately represented. This includes SGM Travellers, migrants, people with disabilities and those who do not publicly identify as LGBTQI+ but may still experience related health inequities. Future research would benefit from explicitly intersectional designs that explore how identities such as ethnicity, class, disability, age and religion shape SGM health experiences. There is also a need to move beyond the dominant focus on mental and sexual health to include social and community wellbeing, ageing, chronic disease and healthcare navigation across the life course.

Many studies in the review relied on convenience sampling, often via online surveys or SGM networks concentrated in urban areas, especially Dublin. Consequently, rural populations, those with lower educational attainment and individuals who are socially or economically marginalised may be underrepresented in the evidence base.

Although our search strategy was broad, our operationalisation of ‘health’ may have constrained the range of included studies. We prioritised peer‐reviewed research focused on physical and mental health, including healthcare access and experience. As a result, social determinants of health, community wellbeing, and broader conceptualisations of health may not have been fully captured.

Moreover, by limiting the review to studies published between 2014 and 2024, we may have missed earlier research with findings that could have contributed further to our understanding of SGM health in Ireland.

Although the timeframe for this review spans the COVID‐19 pandemic, the health impacts of COVID‐19 were not addressed in the studies. This likely reflects a temporal lag between the pandemic period and the publication of peer‐reviewed research, as well as the possibility that COVID‐19 was not a core focus of existing SGM health studies in this context. Nonetheless, the pandemic has had well‐documented effects globally on SGM communities, including exacerbated mental health challenges, reduced access to gender‐affirming care and sexual health services and heightened social isolation (Hong and Skiba 2025; Moreno et al. 2024; Nowaskie and Roesler 2022; Sampogna et al. 2022).

This timeframe was selected as it covered an important period of time during and after major policy advances with regard to sexual and gender minority rights as identified in the introduction.

## 6 Conclusion

The findings from the studies reviewed offer a broad understanding of the current landscape of sexual and gender minority health research in Ireland, but they also expose significant gaps and areas for improvement in both research and service provision. Key thematic domains identified include mental health, sexual health, transgender health, substance use, cancer, older age, happiness and wellbeing and healthcare access. Across these areas, SGM people in Ireland experience significant health inequalities shaped by stigma, discrimination, social exclusion and systemic barriers to care.

The near‐total absence of studies on sexual minority women and intersex individuals underscores the persistent marginalisation within the broader SGM community, both in terms of research focus and healthcare delivery.

This limited research base is not unique to Ireland but reflects wider systemic issues across Europe and the UK, including inadequate integration of SGM health in education and training, gaps in inclusive health policy and a lack of culturally competent specialist services (Harkins and Hoffmann 2024; Hughes et al. 2023; Pfister et al. 2023; Zeeman and Aranda 2020).

Despite significant legislative progress for sexual and gender minorities in Ireland, infrastructural support for SGM health has not kept pace. The invisibility of SGM populations in national health data, the absence of routine SOGI data collection and the urban concentration of services hinder health equity. While Ireland has a vibrant LGBTQI+ civil society, particularly in mental and sexual health advocacy, systemic support for research and service innovation remains limited and inconsistently funded.

Without institutional or national data collection on sexual orientation and gender identity, the true landscape of SGM health remains difficult to assess. Legislative gains, though vital, are not enough. Equitable access to quality healthcare, alongside social, economic and mental health supports, is essential for the full realisation of rights (Gilmore et al. 2024). Legal reforms must be matched by healthcare strategies that address disparities, eliminate discrimination and prioritise the well‐being of SGM people.

Addressing these gaps will require meaningful community engagement, expanded theoretical and methodological approaches, and targeted investment in intersectional, strengths‐based research reflecting the realities of SGM people living in Ireland today.

## 7 Recommendations Based on Findings From This Scoping Review

### 7.1 For Researchers

- Broaden population focus: Include more sexual minority women, non‐binary, transgender, intersex and underrepresented groups (e.g., SGM Travellers, migrants, disabled individuals, rural populations).
- Strengthen intersectional analysis: Systematically collect data on ethnicity, class, disability and location to assess overlapping marginalisations.
- Diversify frameworks: Along with Minority Stress theory, consider alternative approaches to understanding health inequities such as Health Equity Promotion, socio‐ecological models and queer theory.
- Expand research topics: Address gaps in cancer care, ageing, disability and community wellbeing beyond just sexual/mental health.

### 7.2 For Healthcare Practitioners

- Improve competence: Train providers on SGM health, inclusive communication and trans‐affirming care to reduce bias.
- Enhance care environments: Prevent misgendering, identity erasure and poor record‐keeping through updated protocols.

### 7.3 For Policymakers

- Address data gaps: Routinely collect standardised, voluntary SOGI data in health systems.
- Support community partnerships: Fund and involve SGM community groups in health promotion, service design and evaluation.

## Author Contributions

All authors have agreed on the final version and meet at least one of the following criteria (recommended by the ICMJE*): (1) substantial contributions to conception and design, acquisition of data, or analysis and interpretation of data; (2) drafting the article or revising it critically for important intellectual content.

## Conflicts of Interest

The authors declare no conflicts of interest.

## Supporting information


**Data S1:** jan70201‐sup‐0001‐DataS1.pdf.

## Data Availability

The data that support the findings of this study are available from the corresponding author upon reasonable request.
