# Supplementary material for: Scoping Review of Sexual and Gender Minority Health Research in Ireland
Source: J Adv Nurs. 2025 Sep 16;82(5):4625–59. doi: 10.1111/jan.70201 (PMC13069251; doi:10.1111/jan.70201)
Supplement: Supplementary file 1 — Data S1: jan70201‐sup‐0001‐DataS1.pdf. [file JAN-82-4625-s001.pdf]

Supplementary file 2

StudyID (First Author, Year)	Title	Type of Data (i.e. primary, secondary, other such as clinical reports)	Study design (e.g., cross-sectional, longitudinal, qualitative, case study, etc)	Area of Health Research (Sexual Health, HIV, Cancer, Mental Health, Substance Use, General	Study Location(s) (e.g., National - ROI, Dublin, Cork, Galway)	Aim of study	Total sample size (N)	LGBTQ+ population description (e.g., LGBTQ+, sexual minority	Heterosexual comparison group (Y/N)	Include Sexual orientation make-up (e.g. LGB, WSW, etc.) *include percentage/sample size for each	Racial/ethnic make-up *include percentage/sample size of each group*	Age of study sample (include range, mean, and SD—if available)	Method of recruitment of participants (e.g., snowball, National - ROI population survey, health	Primary/independent variables/predictors (include how variables were measured/assessed)	Outcomes measured (include how variables were measured/assessed)	Mediators/moderator (if applicable) (include how variables were measured/assessed)	Other category: qualitative studies (what was being studied? and what type of analysis was used?)	Key findings	Major limitations?	Comments (anything else important to note?)
Barrett (2018)	Drug use among men who have sex with men in Ireland: Prevalence and associated factors from a National - ROI online survey	Primary	Cross-sectional survey	Substance Use	National - ROI	To measure the prevalence of recreational drug use among MSM in a National - ROI	3090	gbMSM	N	Gay/Homosexual = 79.1%; Bisexual = 13.3%; Other = 7.6%	Not specified	<25=31.2%; 25-39=41.3%; 40+=27.5%	National - ROI population survey, convenience sampling	• Age: Continuous variable; categorized <25, 25-39, 40+ years • Education Level: High (degree or higher), Medium (school-leaving), Low	• Any/Recreational Drug Use: Use of specified drugs (excluding poppers) in past year (Yes/No) • Poppers Use: Use of poppers in past	None mentioned	NA	1.High Prevalence of Drug Use: 36% of MSM used recreational drugs, 33% used poppers, and 7% used chemsex drugs in the past year.	1.Cross-Sectional Design: Limits the ability to establish causality between drug use and other factors. 2.Self-Reported Data: Potential for	NA
Bryan (2017)	Supporting LGBT Lives? Complicating the suicide consensus in LGBT mental health research	Primary	Cross-sectional survey and Qualitative study	Mental Health, Suicidality	National - ROI	To ascertain the prevalence of self-harm and suicidality amongst LGBT-identified persons living in Ireland and	1110 (quantitative survey), 40 (qualitative interviews with LGBT people and 14 interviews with service providers)	LGBQ+	N	Gay/Lesbian: 80%, Bisexual: 11%, Questioning: 3%, Heterosexual: <1%, Others: 4%	Not specified	Range: 14-73 years, Mean: 30.5 years	National - ROI population survey, snowball sampling	•Gender Identity: Self-reported in the survey (e.g., male, female, transgender, non-binary). •Sexual Orientation: Self-reported in the	•Suicidal Thoughts: Assessed through self-report indicators. •Suicidal Intent: Assessed through self-report indicators	None mentioned	Experiences of mental health and suicidal distress, thematic and narrative analysis using NVivo	1.Prevalence of Suicidal Thoughts and Actions: Majority of respondents never (42%) or only rarely (32%) considered suicide. Among younger participants	1.Cross-Sectional Design: Limits ability to establish causality and determine temporal relationships between factors and outcomes	NA
Carey (2023)	Factors associated with lower knowledge of HIV and STI transmission, testing and treatment among MSM in Ireland: findings from the MSM Internet Survey Ireland (MISI) 2015	Primary	Cross-sectional survey	Sexual Health, HIV	National - ROI	To identify factors associated with lower knowledge of HIV and STI transmission, testing, and treatment among	2905	gbMSM	N	Predominantly gay, some bisexual and straight/other	Not specified	Range: 18-78 years, Median: 30 years; [illegible]	National - ROI population survey, convenience sampling	•Age, •education, •area of residence, •employment	Lower knowledge of HIV/STI transmission, testing, and treatment (measured using 13 factual statements)	None mentioned	NA	1.36% had lower knowledge 2.Younger MSM (18-24) had lower knowledge (aOR 1.98) 3.MSM with less than a degree had	1.Cross-Sectional Design: Limits the ability to establish causality and cannot draw temporal relationships. 2.Self-Reported Data: May introduce	NA
Ceatha (2016)	Mastering wellness: LGBT peopleâ€™s understanding of wellbeing through interest sharing	Primary	Qualitative	Mental Health, Wellbeing	Dublin, Ireland	To explore LGBT people's understanding of their wellbeing through interest sharing	11	LGBTQ+	N	Lesbian: 5, Gay: 4, Bisexual: 1, Transgender: 1	9=Irish, 1=European, 1=North American	Range: 22-56 years	Purposive sampling	NA	NA	NA	In-depth interviews regarding background, joining groups, wellness, and interests, thematic analysis	1.Mastering Wellness: Participants described mastering wellness through active engagement in interest groups, emphasizing personal agency and	1.Small sample size limits generalisability 2.Over-representation of educated professionals	NA
Ceatha (2019)	The Power of Recognition: A Qualitative Study of Social Connectedness and Wellbeing through LGBT Sporting, Creative and Social Groups in Ireland	Primary	Qualitative	Mental Health, Wellbeing	Dublin, Ireland	To explore the social meaning and significance of LGBT social and community through their sporting	11	LGBTQ+	N	Lesbian: 5, Gay: 4, Bisexual: 1, Transgender: 1	9=Irish, 1=European, 1=North American	Range: 22-56 years	Purposive sampling	NA	NA	NA	In-depth interviews regarding background, joining groups, wellness, and interests, thematic analysis	1.The Sample: Included 11 participants (5 lesbian women, 4 gay men, 1 bisexual person, 1 transgender person) aged 22 to 56, involved in various LGBT and non-	1.Small Sample Size: The study involved only 11 participants, which limits the generalisability of the findings to other LGBT populations	NA
Connolly (2018)	Is being gay bad for your health and wellbeing? Cultural issues affecting gay men accessing and using health services in the Republic of Ireland	Primary	Qualitative	General Health/Wellbeing	Republic of Ireland (primarily on the east coast, including Dublin and Cork)	To explore how the sexual orientation of gay men creates inequalities in the type of care delivered to them	20	Gay Men	N	100% gay men	Not specified	Range: 24-60 years	Snowball sampling	NA	NA	NA	Explored the cultural domain and health inequalities through semi-structured interviews and biographical questionnaires	1.Being gay in the Republic of Ireland: Persistent cultural challenges and homophobia despite legal progress. 2.Being gay: Attitudes of the Catholic	1.Sample size: The study's small sample size (20 men) limits generalisability to the broader population. 2.Demographic bias: Predominantly	NA
Cotter (2014)	Victimisation and psychosocial difficulties associated with sexual orientation concerns: a school-based study of adolescents	Primary	Cross-sectional survey	Mental Health, Substance Use, General Health/Wellbeing	Cork and Kerry, Ireland	To examine the association between sexual orientation concerns, mental health difficulties, and suicidal and	1112	LGB	Y	58 students with sexual orientation concerns (35 boys, 23 girls; mean age 14.1 years)	Not specified	Range: 13-16 years; Mean: 13.7 years	Random selection from mixed-gender secondary schools	Sexual orientation concerns (measured by self-report questionnaire)	Victimisation, substance misuse, mental health difficulties, suicidal behaviour, relationships, sexual activity (measured by standardised scales and self-report	None mentioned	NA	1.Increased Victimization: Adolescents with sexual orientation concerns faced significantly higher levels of victimization. They were five times more likely to have	1.Small Sample Size: The group with sexual orientation concerns was small (58 students), limiting the ability to generalise findings	NA
Cox (2017)	Gardnerella vaginalis and Mollicute detection in rectal swabs from men who have sex with men	Primary	Cross-sectional survey	Sexual Health, HIV, General Health/Wellbeing	Belfast, Northern Ireland	To assess the prevalence and bacterial load of Gardnerella vaginalis and Mollicutes in rectal swabs	107	gbMSM	N	Not specified	Not specified	Mean age: 35.3 years; Standard Deviation: 12.5 years	Convenience sampling for those attending sexual health clinic	Presence of Gardnerella vaginalis and Mollicutes (measured by qPCR)	Bacterial prevalence, bacterial load, co-infections, and synergies between bacteria (measured by qPCR and statistical analysis)	None mentioned	NA	1.High Prevalence of G. vaginalis: Gardnerella vaginalis was found in 83.2% of rectal swabs from MSM, indicating common presence or repeated exposure	1.Small Sample Size: The study included only 107 rectal swabs, limiting the generalisability of the findings. 2.Lack of Clinical Data: Anonymised	NA
Cullinan (2022)	A cross-sectional analysis of psychological distress among higher education students in Ireland	Primary	Cross-sectional survey	Mental Health, General Health/Wellbeing	National - ROI	To examine levels of [illegible]	5201	LGBTQ+	Y	Heterosexual: 85.87% (4466 students) [illegible]	Not specified	18-19 years: 23.01% [illegible]	Convenience sampling	Age, gender, sexual orientation, country [illegible]	Levels of depression, anxiety, and stress [illegible]	None mentioned	NA	1.High Prevalence of Psychological [illegible]	1.Selection Bias: The self-selection [illegible]	NA
Daly (2021)	Potential alcohol use disorder among MSM in Ireland - findings from the European MSM internet survey (EMIS 2017)	Primary	[illegible]	[illegible]	[illegible]	[illegible]	[illegible]	[illegible]	[illegible]	[illegible]	[illegible]	[illegible]	[illegible]	[illegible]	[illegible]	[illegible]	[illegible]	[illegible]	[illegible]	[illegible]
Daly (2023)	Recreational and sexualised drug use among gay, bisexual, and other men who have sex with men (gbMSM) in Ireland-Findings from the European MSM internet survey (EMIS) 2017	Primary	[illegible]	[illegible]	[illegible]	[illegible]	[illegible]	[illegible]	[illegible]	[illegible]	[illegible]	[illegible]	[illegible]	[illegible]	[illegible]	[illegible]	[illegible]	[illegible]	[illegible]	[illegible]
de Vries (2020)	An exploration of happiness within the Irish LGBTI community	[illegible]	[illegible]	[illegible]	[illegible]	[illegible]	[illegible]	[illegible]	[illegible]	[illegible]	[illegible]	[illegible]	[illegible]	[illegible]	[illegible]	[illegible]	[illegible]	[illegible]	[illegible]	[illegible]
de Vries (2023)	An exploration of mental distress in transgender people in Ireland with reference to minority stress and [illegible]	[illegible]	[illegible]	[illegible]	[illegible]	[illegible]	[illegible]	[illegible]	[illegible]	[illegible]	[illegible]	[illegible]	[illegible]	[illegible]	[illegible]	[illegible]	[illegible]	[illegible]	[illegible]	[illegible]
Delaney (2021)	A phenomenological exploration of transgender peopleâ€™s experiences of mental health services in Ireland	[illegible]	[illegible]	[illegible]	[illegible]	[illegible]	[illegible]	[illegible]	[illegible]	[illegible]	[illegible]	[illegible]	[illegible]	[illegible]	[illegible]	[illegible]	[illegible]	[illegible]	[illegible]	[illegible]
Dolphin (2018)	Risky sex behaviours among college students: The psychosocial profile	[illegible]	[illegible]	[illegible]	[illegible]	[illegible]	[illegible]	[illegible]	[illegible]	[illegible]	[illegible]	[illegible]	[illegible]	[illegible]	[illegible]	[illegible]	[illegible]	[illegible]	[illegible]	[illegible]
Glynn (2018)	Chemsex, risk behaviours and sexually transmitted infections among men who have sex with men in Dublin [illegible]	[illegible]	[illegible]	[illegible]	[illegible]	[illegible]	[illegible]	[illegible]	[illegible]	[illegible]	[illegible]	[illegible]	[illegible]	[illegible]	[illegible]	[illegible]	[illegible]	[illegible]	[illegible]	[illegible]
Higgins (2016)	Building resilience in the face of adversity: navigation processes used by older lesbian, gay, bisexual and [illegible]	[illegible]	[illegible]	[illegible]	[illegible]	[illegible]	[illegible]	[illegible]	[illegible]	[illegible]	[illegible]	[illegible]	[illegible]	[illegible]	[illegible]	[illegible]	[illegible]	[illegible]	[illegible]	[illegible]
Higgins (2021)	LGBT+ young peopleâ€™s perceptions of barriers to accessing mental health services in Ireland	[illegible]	[illegible]	[illegible]	[illegible]	[illegible]	[illegible]	[illegible]	[illegible]	[illegible]	[illegible]	[illegible]	[illegible]	[illegible]	[illegible]	[illegible]	[illegible]	[illegible]	[illegible]	[illegible]
Horgan (2018)	Depressive symptoms and suicidal ideation among Irish undergraduate college students	[illegible]	[illegible]	[illegible]	[illegible]	[illegible]	[illegible]	[illegible]	[illegible]	[illegible]	[illegible]	[illegible]	[illegible]	[illegible]	[illegible]	[illegible]	[illegible]	[illegible]	[illegible]	[illegible]
Howell (2019)	Seeking help when transgender: Exploring the difference in mental and physical health seeking behaviors [illegible]	[illegible]	[illegible]	[illegible]	[illegible]	[illegible]	[illegible]	[illegible]	[illegible]	[illegible]	[illegible]	[illegible]	[illegible]	[illegible]	[illegible]	[illegible]	[illegible]	[illegible]	[illegible]	[illegible]
Joyce (2018)	The experience and meaning of problematic â€˜GHB/GBLâ€™ use in an Irish context: An interpretative [illegible]	[illegible]	[illegible]	[illegible]	[illegible]	[illegible]	[illegible]	[illegible]	[illegible]	[illegible]	[illegible]	[illegible]	[illegible]	[illegible]	[illegible]	[illegible]	[illegible]	[illegible]	[illegible]	[illegible]
Judge (2014)	Gender dysphoria - prevalence and co-morbidities in an irish adult population	[illegible]	[illegible]	[illegible]	[illegible]	[illegible]	[illegible]	[illegible]	[illegible]	[illegible]	[illegible]	[illegible]	[illegible]	[illegible]	[illegible]	[illegible]	[illegible]	[illegible]	[illegible]	[illegible]
Keane (2022)	Evaluation of the impact of human immunodeficiency virus pre-exposure prophylaxis on new human immuno[illegible]	[illegible]	[illegible]	[illegible]	[illegible]	[illegible]	[illegible]	[illegible]	[illegible]	[illegible]	[illegible]	[illegible]	[illegible]	[illegible]	[illegible]	[illegible]	[illegible]	[illegible]	[illegible]	[illegible]
Kearns (2023)	Transgender and non-binary demographics, referrals, and comorbidities among young Irish adults (2016-[illegible]	[illegible]	[illegible]	[illegible]	[illegible]	[illegible]	[illegible]	[illegible]	[illegible]	[illegible]	[illegible]	[illegible]	[illegible]	[illegible]	[illegible]	[illegible]	[illegible]	[illegible]	[illegible]	[illegible]
Kearns (2024)	Factors that help and hinder transgender and nonbinary youth accessing gender care in Ireland: A multista[illegible]	[illegible]	[illegible]	[illegible]	[illegible]	[illegible]	[illegible]	[illegible]	[illegible]	[illegible]	[illegible]	[illegible]	[illegible]	[illegible]	[illegible]	[illegible]	[illegible]	[illegible]	[illegible]	[illegible]
Keaveney (2014)	High prevalence of asymptomatic sexually transmitted infections in HIV-infected men who have sex with me[illegible]	[illegible]	[illegible]	[illegible]	[illegible]	[illegible]	[illegible]	[illegible]	[illegible]	[illegible]	[illegible]	[illegible]	[illegible]	[illegible]	[illegible]	[illegible]	[illegible]	[illegible]	[illegible]	[illegible]
Kerr (2022)	Changing demographics and immunity to vaccine preventable diseases in people with HIV in Ireland	[illegible]	[illegible]	[illegible]	[illegible]	[illegible]	[illegible]	[illegible]	[illegible]	[illegible]	[illegible]	[illegible]	[illegible]	[illegible]	[illegible]	[illegible]	[illegible]	[illegible]	[illegible]	[illegible]
Kerrigan (2019)	'Ghosts from the past': The re-emergence of internalized religious stigma following diagnosis of HIV [illegible]	[illegible]	[illegible]	[illegible]	[illegible]	[illegible]	[illegible]	[illegible]	[illegible]	[illegible]	[illegible]	[illegible]	[illegible]	[illegible]	[illegible]	[illegible]	[illegible]	[illegible]	[illegible]	[illegible]
Költő (2021)	Connected, Respected and Contributing to Their World: The Case of Sexual Minority and Non-Minority Yo[illegible]	[illegible]	[illegible]	[illegible]	[illegible]	[illegible]	[illegible]	[illegible]	[illegible]	[illegible]	[illegible]	[illegible]	[illegible]	[illegible]	[illegible]	[illegible]	[illegible]	[illegible]	[illegible]	[illegible]
Költő (2023)	Perceived Discrimination Among Adolescents in Ireland	[illegible]	[illegible]	[illegible]	[illegible]	[illegible]	[illegible]	[illegible]	[illegible]	[illegible]	[illegible]	[illegible]	[illegible]	[illegible]	[illegible]	[illegible]	[illegible]	[illegible]	[illegible]	[illegible]
Lehmann (2020)	Autism trait prevalence in treatment seeking adolescents and adults attending specialist gender services	[illegible]	[illegible]	[illegible]	[illegible]	[illegible]	[illegible]	[illegible]	[illegible]	[illegible]	[illegible]	[illegible]	[illegible]	[illegible]	[illegible]	[illegible]	[illegible]	[illegible]	[illegible]	[illegible]
Lehmann (2021)	Dramaturgical accounts of transgender individuals: Impression management in the presentation of self to [illegible]	[illegible]	[illegible]	[illegible]	[illegible]	[illegible]	[illegible]	[illegible]	[illegible]	[illegible]	[illegible]	[illegible]	[illegible]	[illegible]	[illegible]	[illegible]	[illegible]	[illegible]	[illegible]	[illegible]
Losty (2018)	Falling outside of the â€˜nice little binary boxâ€™: A psychoanalytic exploration of the non-binary gender id[illegible]	[illegible]	[illegible]	[illegible]	[illegible]	[illegible]	[illegible]	[illegible]	[illegible]	[illegible]	[illegible]	[illegible]	[illegible]	[illegible]	[illegible]	[illegible]	[illegible]	[illegible]	[illegible]	[illegible]
Mahon (2022a)	Mental, physical and sexual health in sexual minority third-level students: Findings from my world survey [illegible]	[illegible]	[illegible]	[illegible]	[illegible]	[illegible]	[illegible]	[illegible]	[illegible]	[illegible]	[illegible]	[illegible]	[illegible]	[illegible]	[illegible]	[illegible]	[illegible]	[illegible]	[illegible]	[illegible]
Mahon (2022b)	An exploratory investigation of body esteem, body dissatisfaction and body change behaviours in sexual mi[illegible]	[illegible]	[illegible]	[illegible]	[illegible]	[illegible]	[illegible]	[illegible]	[illegible]	[illegible]	[illegible]	[illegible]	[illegible]	[illegible]	[illegible]	[illegible]	[illegible]	[illegible]	[illegible]	[illegible]
Mahon (2021)	Risk and protective factors for social anxiety among sexual minority individuals	[illegible]	[illegible]	[illegible]	[illegible]	[illegible]	[illegible]	[illegible]	[illegible]	[illegible]	[illegible]	[illegible]	[illegible]	[illegible]	[illegible]	[illegible]	[illegible]	[illegible]	[illegible]	[illegible]
McCann (2015)	People who are transgender: Mental health concerns	[illegible]	[illegible]	[illegible]	[illegible]	[illegible]	[illegible]	[illegible]	[illegible]	[illegible]	[illegible]	[illegible]	[illegible]	[illegible]	[illegible]	[illegible]	[illegible]	[illegible]	[illegible]	[illegible]
McCann (2019)	Education and practice developments: Addressing the psychosocial concerns and support needs of LGBT+ [illegible]	[illegible]	[illegible]	[illegible]	[illegible]	[illegible]	[illegible]	[illegible]	[illegible]	[illegible]	[illegible]	[illegible]	[illegible]	[illegible]	[illegible]	[illegible]	[illegible]	[illegible]	[illegible]	[illegible]
McCann (2020a)	Psychosocial needs of bisexual people: Findings from a mixed-methods research study	[illegible]	[illegible]	[illegible]	[illegible]	[illegible]	[illegible]	[illegible]	[illegible]	[illegible]	[illegible]	[illegible]	[illegible]	[illegible]	[illegible]	[illegible]	[illegible]	[illegible]	[illegible]	[illegible]
McCann (2020b)	The views and experiences of lesbians regarding their mental health needs and concerns: Qualitative findi[illegible]	[illegible]	[illegible]	[illegible]	[illegible]	[illegible]	[illegible]	[illegible]	[illegible]	[illegible]	[illegible]	[illegible]	[illegible]	[illegible]	[illegible]	[illegible]	[illegible]	[illegible]	[illegible]	[illegible]
McCann (2014a)	Challenges to and opportunities for improving mental health services for lesbian, gay, bisexual, and transge[illegible]	[illegible]	[illegible]	[illegible]	[illegible]	[illegible]	[illegible]	[illegible]	[illegible]	[illegible]	[illegible]	[illegible]	[illegible]	[illegible]	[illegible]	[illegible]	[illegible]	[illegible]	[illegible]	[illegible]
McCann (2014b)	Survey of lesbian, gay, bisexual, and transgender people's experiences of mental health services in Ireland	[illegible]	[illegible]	[illegible]	[illegible]	[illegible]	[illegible]	[illegible]	[illegible]	[illegible]	[illegible]	[illegible]	[illegible]	[illegible]	[illegible]	[illegible]	[illegible]	[illegible]	[illegible]	[illegible]
McConkey (2018)	Exploring the lived experience of gay men with prostate cancer: A phenomenological study	[illegible]	[illegible]	[illegible]	[illegible]	[illegible]	[illegible]	[illegible]	[illegible]	[illegible]	[illegible]	[illegible]	[illegible]	[illegible]	[illegible]	[illegible]	[illegible]	[illegible]	[illegible]	[illegible]
McLafferty (2017)	Mental health, behavioural problems and treatment seeking among students commencing university in Nort[illegible]	[illegible]	[illegible]	[illegible]	[illegible]	[illegible]	[illegible]	[illegible]	[illegible]	[illegible]	[illegible]	[illegible]	[illegible]	[illegible]	[illegible]	[illegible]	[illegible]	[illegible]	[illegible]	[illegible]
Moloney (2021)	Assessing the Quality of Care Delivered to Transgender and Gender Diverse Patients with Cancer in Irela[illegible]	[illegible]	[illegible]	[illegible]	[illegible]	[illegible]	[illegible]	[illegible]	[illegible]	[illegible]	[illegible]	[illegible]	[illegible]	[illegible]	[illegible]	[illegible]	[illegible]	[illegible]	[illegible]	[illegible]
Murphy (2015)	Optimism, community attachment and serostatus disclosure among HIV-positive men who have sex with me[illegible]	[illegible]	[illegible]	[illegible]	[illegible]	[illegible]	[illegible]	[illegible]	[illegible]	[illegible]	[illegible]	[illegible]	[illegible]	[illegible]	[illegible]	[illegible]	[illegible]	[illegible]	[illegible]	[illegible]
Murphy (2016)	Serostatus disclosure, stigma resistance, and identity management among HIV-Positive gay men in Ireland	[illegible]	[illegible]	[illegible]	[illegible]	[illegible]	[illegible]	[illegible]	[illegible]	[illegible]	[illegible]	[illegible]	[illegible]	[illegible]	[illegible]	[illegible]	[illegible]	[illegible]	[illegible]	[illegible]
Nic Lochlainn (2017)	Using data from a behavioural survey of men who have sex with men (MSM) to estimate the number likely [illegible]	[illegible]	[illegible]	[illegible]	[illegible]	[illegible]	[illegible]	[illegible]	[illegible]	[illegible]	[illegible]	[illegible]	[illegible]	[illegible]	[illegible]	[illegible]	[illegible]	[illegible]	[illegible]	[illegible]
O'Connell (2017)	Late HIV presentation - missed opportunities and factors associated with a changing pattern over time	[illegible]	[illegible]	[illegible]	[illegible]	[illegible]	[illegible]	[illegible]	[illegible]	[illegible]	[illegible]	[illegible]	[illegible]	[illegible]	[illegible]	[illegible]	[illegible]	[illegible]	[illegible]	[illegible]
O'Connor (2019)	Use of geosocial networking applications is independently associated with diagnosis of STI among men wh[illegible]	[illegible]	[illegible]	[illegible]	[illegible]	[illegible]	[illegible]	[illegible]	[illegible]	[illegible]	[illegible]	[illegible]	[illegible]	[illegible]	[illegible]	[illegible]	[illegible]	[illegible]	[illegible]	[illegible]
O'Donnell (2019)	Inequalities in HIV testing uptake and needs among men who have sex with men living in Ireland: findings f[illegible]	[illegible]	[illegible]	[illegible]	[illegible]	[illegible]	[illegible]	[illegible]	[illegible]	[illegible]	[illegible]	[illegible]	[illegible]	[illegible]	[illegible]	[illegible]	[illegible]	[illegible]	[illegible]	[illegible]
O'Rourke (2021)	Seroprevalence of human herpesvirus 8 in Ireland among blood donors, men who have sex with men, and he[illegible]	[illegible]	[illegible]	[illegible]	[illegible]	[illegible]	[illegible]	[illegible]	[illegible]	[illegible]	[illegible]	[illegible]	[illegible]	[illegible]	[illegible]	[illegible]	[illegible]	[illegible]	[illegible]	[illegible]
Robinson (2019)	Integration of a recent infection testing algorithm into HIV surveillance in Ireland: improving HIV knowledge [illegible]	[illegible]	[illegible]	[illegible]	[illegible]	[illegible]	[illegible]	[illegible]	[illegible]	[illegible]	[illegible]	[illegible]	[illegible]	[illegible]	[illegible]	[illegible]	[illegible]	[illegible]	[illegible]	[illegible]
Saab (2023)	Promoting â€˜testicular awarenessâ€™: Co-creation of an inclusive campaign using the world cafÃ© met[illegible]	[illegible]	[illegible]	[illegible]	[illegible]	[illegible]	[illegible]	[illegible]	[illegible]	[illegible]	[illegible]	[illegible]	[illegible]	[illegible]	[illegible]	[illegible]	[illegible]	[illegible]	[illegible]	[illegible]
Sadlier (2016)	HPV vaccine acceptability in HIV-infected and HIV negative men who have sex with men (MSM) in Ireland	[illegible]	[illegible]	[illegible]	[illegible]	[illegible]	[illegible]	[illegible]	[illegible]	[illegible]	[illegible]	[illegible]	[illegible]	[illegible]	[illegible]	[illegible]	[illegible]	[illegible]	[illegible]	[illegible]
Sadlier (2014)	Prevalence of human papillomavirus in men who have sex with men in the era of an effective vaccine; a call [illegible]	[illegible]	[illegible]	[illegible]	[illegible]	[illegible]	[illegible]	[illegible]	[illegible]	[illegible]	[illegible]	[illegible]	[illegible]	[illegible]	[illegible]	[illegible]	[illegible]	[illegible]	[illegible]	[illegible]
Sharek (2015)	Older LGBT people's experiences and concerns with healthcare professionals and services in Ireland	[illegible]	[illegible]	[illegible]	[illegible]	[illegible]	[illegible]	[illegible]	[illegible]	[illegible]	[illegible]	[illegible]	[illegible]	[illegible]	[illegible]	[illegible]	[illegible]	[illegible]	[illegible]	[illegible]
Travers (2020)	Lesbian, gay or bisexual identity as a risk factor for trauma and mental health problems in Northern Irish stu[illegible]	[illegible]	[illegible]	[illegible]	[illegible]	[illegible]	[illegible]	[illegible]	[illegible]	[illegible]	[illegible]	[illegible]	[illegible]	[illegible]	[illegible]	[illegible]	[illegible]	[illegible]	[illegible]	[illegible]
Van Hout (2019)	Chasing the rainbow: Pleasure, sex-based sociality and consumerism in navigating and exiting the Irish Ch[illegible]	[illegible]	[illegible]	[illegible]	[illegible]	[illegible]	[illegible]	[illegible]	[illegible]	[illegible]	[illegible]	[illegible]	[illegible]	[illegible]	[illegible]	[illegible]	[illegible]	[illegible]	[illegible]	[illegible]
White (2023)	Hepatitis A and B vaccination in gbMSM in Ireland: findings from the European MSM Internet Survey 2017	[illegible]	[illegible]	[illegible]	[illegible]	[illegible]	[illegible]	[illegible]	[illegible]	[illegible]	[illegible]	[illegible]	[illegible]	[illegible]	[illegible]	[illegible]	[illegible]	[illegible]	[illegible]	[illegible]
